# The brown algal mode of tip growth: Keeping stress under control

**DOI:** 10.1371/journal.pbio.2005258

**Published:** 2019-01-14

**Authors:** Hervé Rabillé, Bernard Billoud, Benoit Tesson, Sophie Le Panse, Élodie Rolland, Bénédicte Charrier

**Affiliations:** 1 CNRS, Sorbonne Université, Morphogenesis of Macro Algae, UMR8227, Station Biologique, Roscoff, France; 2 SCRIPPS Institution of Oceanography, University of California, San Diego, San Diego, California, United States of America; 3 MerImage platform, FR2424, CNRS, Sorbonne Université, Station Biologique, Roscoff, France; Cornell University, United States of America

## Abstract

Tip growth has been studied in pollen tubes, root hairs, and fungal and oomycete hyphae and is the most widely distributed unidirectional growth process on the planet. It ensures spatial colonization, nutrient predation, fertilization, and symbiosis with growth speeds of up to 800 μm h^−1^. Although turgor-driven growth is intuitively conceivable, a closer examination of the physical processes at work in tip growth raises a paradox: growth occurs where biophysical forces are low, because of the increase in curvature in the tip. All tip-growing cells studied so far rely on the modulation of cell wall extensibility via the polarized excretion of cell wall–loosening compounds at the tip. Here, we used a series of quantitative measurements at the cellular level and a biophysical simulation approach to show that the brown alga *Ectocarpus* has an original tip-growth mechanism. In this alga, the establishment of a steep gradient in cell wall thickness can compensate for the variation in tip curvature, thereby modulating wall stress within the tip cell. Bootstrap analyses support the robustness of the process, and experiments with fluorescence recovery after photobleaching (FRAP) confirmed the active vesicle trafficking in the shanks of the apical cell, as inferred from the model. In response to auxin, biophysical measurements change in agreement with the model. Although we cannot strictly exclude the involvement of a gradient in mechanical properties in *Ectocarpus* morphogenesis, the viscoplastic model of cell wall mechanics strongly suggests that brown algae have evolved an alternative strategy of tip growth. This strategy is largely based on the control of cell wall thickness rather than fluctuations in cell wall mechanical properties.

## Author summary

Tip growth is known in organisms with filament-like structures, such as fungi (e.g., hyphae), plants (e.g., root hairs, moss protonemata), and algae (e.g., filamentous thalli). The driving force for growth in these organisms is the difference in osmotic pressure (turgor) between the inside of the cell and the external medium, a force contained by the cell wall. Physical laws imply that the higher the curvature of the cell, the lower the pressure (stress) perceived by the cell wall. Paradoxically, growth takes place at the dome-shaped cell apex, which has high curvature. Tip-growing cells studied so far (mainly plants) compensate the low wall stress in the apex by chemically loosening their cell wall. We studied *Ectocarpus*, which is a representative of brown algae, a eukaryotic branch very divergent from land plants, fungi, and green algae. We carried out a series of quantitative measurements at the cellular level and showed that the cell wall is thinner at the tip (36 nm) than on the shanks (170 to 500 nm). Using a viscoplastic model of cell wall expansion, we showed that the cell wall thickness gradient, together with dome curvature, generates sufficient wall stress to account for the observed growth pattern.

**Fig 1:**
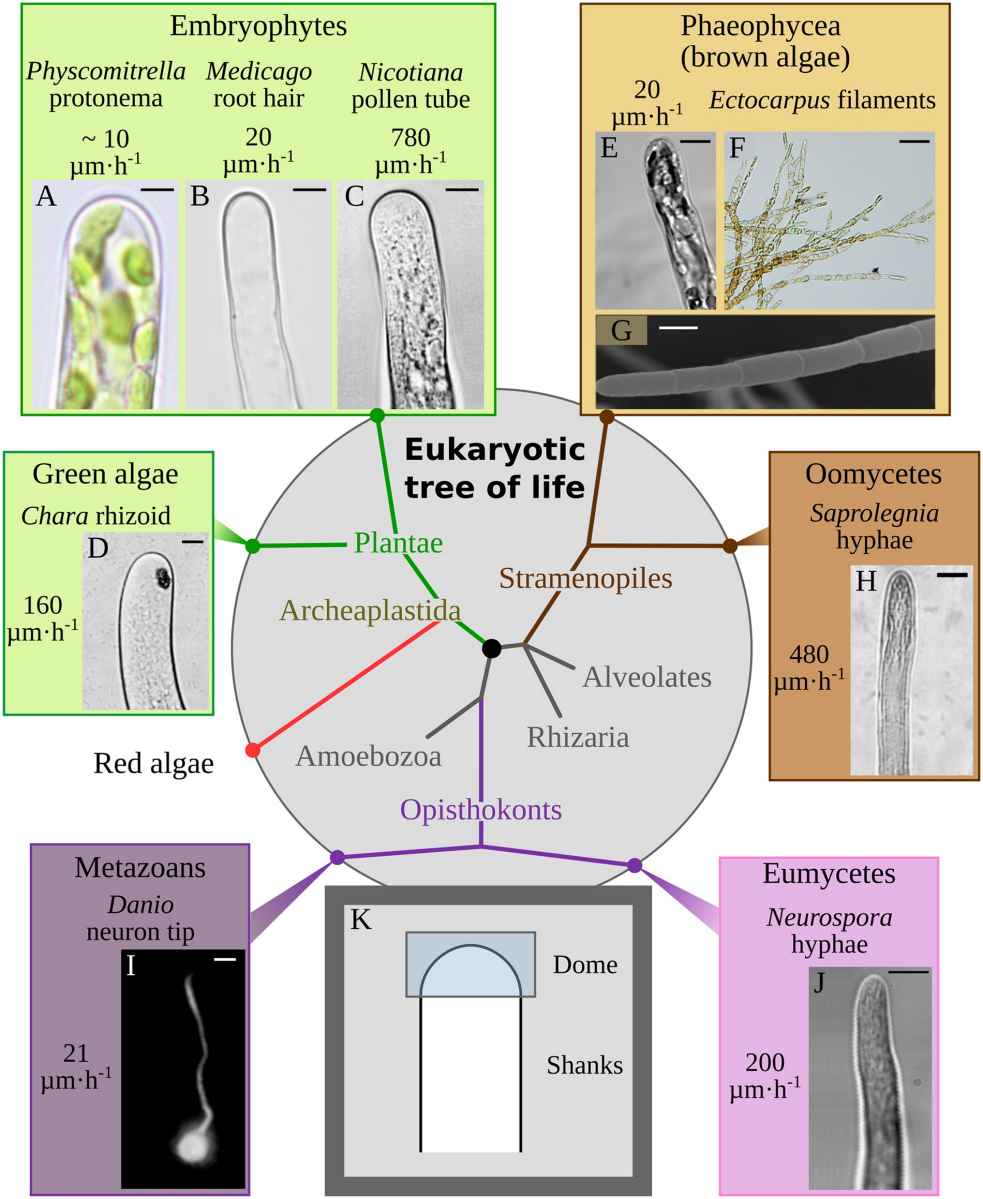
Diversity of tip growth in the eukaryotic tree of life. Phylogenetic position of eukaryotic taxa with tip-growing organisms. Cell shapes and growth rates are shown. (A–D) Archaeplastida group. (A) Moss protonema; (B) root hair; (C) pollen tube; (D) green algal filament. (E–H) Stramenopiles, which include the coenocytic oomycetes and the multicellular brown algae, e.g., the filamentous alga *Ectocarpus*. (E) *Ectocarpus* apical cell of a prostrate sporophyte filament; (F) *Ectocarpus* tuft with several branches; (G) *Ectocarpus* filament viewed with an SEM; (H) oomycete hyphae. (I, J) Tip growth in the Opisthokonta group. (I) Neurons of metazoans; (J) fungal hyphae. (K) Two main cellular regions defining tip-growing cells. Top frames are the two taxa compared in this study (pollen tube and brown algal filament). Bar = 5 μm (A–C, E, H–J), 10 μm (G), 20 μm (D, F). *Photo credits*: *(A) A*. *Le Bail*, *Erlangen University*, *Germany; (B) Florian Frugier*, *IPS2 Gif/Yvette*, *France*, *(C) B*. *Kost*, *Erlangen University*, *Germany; (D) M*. *Braun*, *Erlangen University; (G) A*. *Le Bail*, *Station Biologique Roscoff CNRS-UPMC*, *France*, *(H) reproduced with permission from* Cell Research [8]; *(I) reproduced with permission from* Disease Models Mechanisms (*CC-BY license*) [9]; *(J) reproduced with permission from* Journal of Cell Science [10]. SEM, scanning electronic microscope.

In multicellular organisms, morphogenesis—from the cell to the organ level—relies largely on mechanical processes [1,2]. Cell expansion results from the balance between forces promoting extension (turgor, cytoskeleton) and structural resistance to deformation (cytoskeleton, cell wall, plasma membrane, and cytoplasm). Tip growth is one of the simplest cases of cell morphogenesis, characterized by pronounced cell polarization ensuring unidirectional exploration and colonization of the surrounding space through the expansion of the most distal region of the cell: the tip. It is encountered in many eukaryotes throughout the tree of life [3], with a diversity of cell shapes [4] and growth rates (from 2.5 to 800 μm h^−1^) [5–7] (Fig 1).

Physical laws imply that wall stress (σ, force per unit of area) due to internal pressure is lower in the tip of the cell than in the shanks of the cell, because the cell wall curvature increases in the dome-shaped tip [11]. Beyond the apparently simple process of shifting material to the cell front, a wealth of mechanical, cellular, and chemical mechanisms are required to ensure growth where wall stress is low and to prevent it where wall stress is high.

In plant and fungi, the cell wall is the most resistant compartment of the cell. Fungal hyphae and tip cells of land plants (e.g., pollen tubes and root hairs) secrete cell wall–loosening factors together with cell wall–building components, making cell walls susceptible to stretch despite the low wall stress at the tip, whereas the more proximal cell wall in the shanks becomes stiffer, resisting the higher wall stress [12,13]. To explore potential mechanistic conservation or alternative strategies, we studied tip growth in the model brown alga *Ectocarpus* [14], which belongs to the Stramenopiles, a distinct branch of the domain Eukaryota [15] (Fig 1). Brown algae are multicellular organisms. They can be microscopic or as large as land plants (up to 40 m) and are harvested for human subsistence and activities [16]. Their relatively recent emergence in the tree of life (approximately 200 million years [My]) [17]—compared with land plants (450 My) [18], green algae (750 My) [19], red algae (approximately 1.2 billion years [By]) [20], and metazoans (approximately 600 My) [21]—occurred independently of the other multicellular organisms [15]. In addition, their growth in marine environments (high salt concentration, high external pressure, and reduced perception of gravitational forces compared with those on land) raises further questions about the physical forces these organisms rely on to grow. Previous studies have illustrated the uniqueness of these organisms regarding their energy and primary metabolisms [22], their cellular structural components [23,24], and their genetic features [25]. *Ectocarpus* has emerged as a great model for brown algae over the past 15 y [14,25]. It is a tiny uniseriate filamentous brown alga (Fig 1E, 1F and 1G) with low body complexity. Furthermore, its filament cells are easy to observe and handle (e.g., laser capture microdissection [26] or atomic force microscopy [AFM] [27]), making *Ectocarpus* particularly amenable to sophisticated fundamental studies in cellular and developmental biology. Zygote germination, filament growth, and subsequent branching occur via tip growth [28], and resulting apical cells exert both growing and branching negative controls on subapical tissues [29], making apical cells key organizing centers for further development.

In this article, we characterized a biophysical mechanism able to account for tip growth in *Ectocarpus* apical cells and compared it with a representative of other eukaryotic tip-growing cells: the pollen tube. Data on growth location and direction, wall stress, and cell wall structure are all prerequisites for the accurate use of biophysical models of tip growth. Using a viscoplastic model of the cell wall and biophysical data specific to *Ectocarpus* apical cells, we demonstrate a gradient in wall stress, which provides ample support for an alternative pattern of tip growth.

## Results

### Growth takes place in the apical dome and is orthogonal to the cell surface

**Fig 2:**
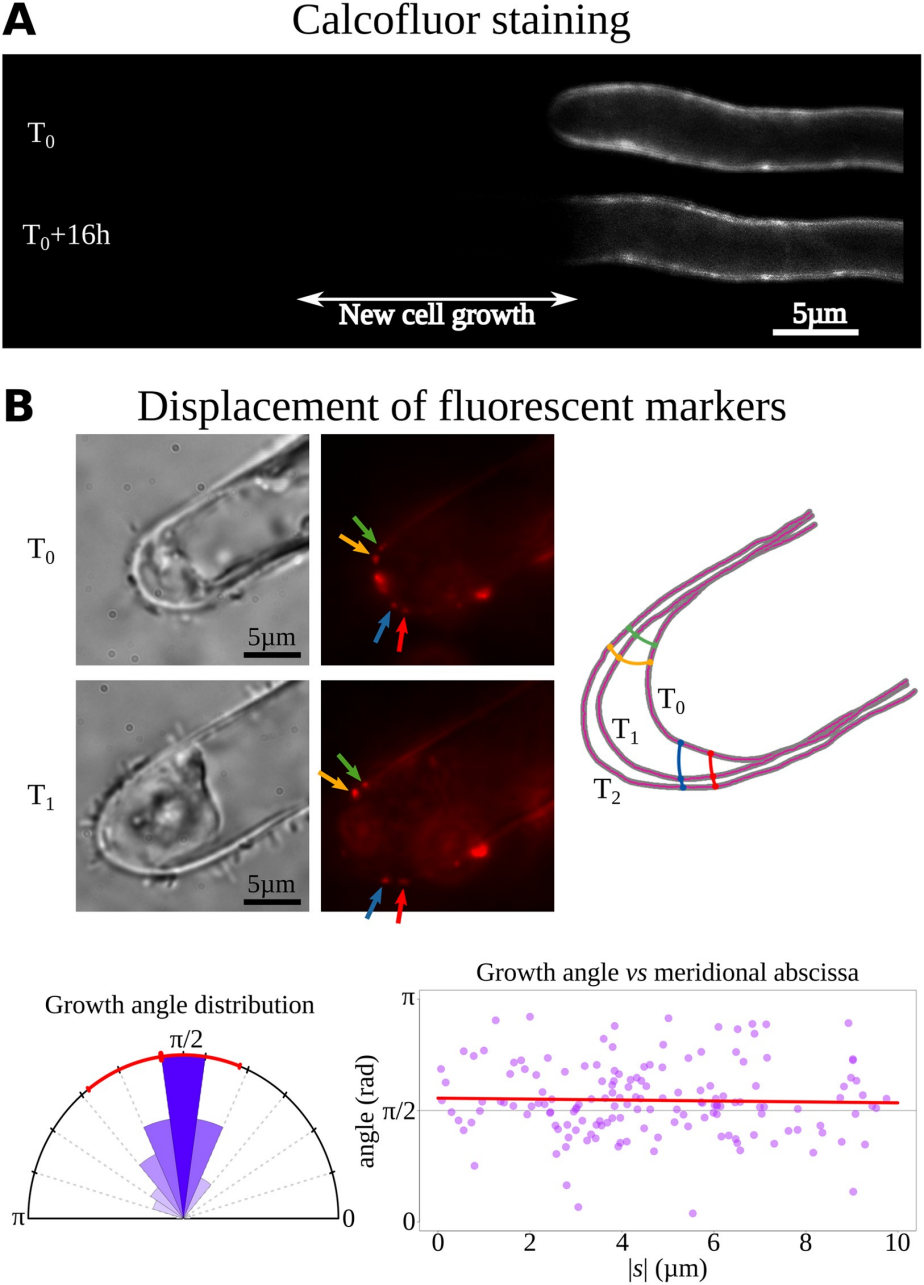
Position and direction of cell wall expansion during growth. (A) Pulse-chase experiment using calcofluor-white stain during growth. Filaments were washed to remove the calcofluor-white immediately after staining and observed again after 16 h. The dark area thus corresponds to recently deposited cell materials. (B) Orthogonal growth in the apical cell. (Top) Cell wall deformation at the apex of an apical cell during growth, as visualized by the displacement of fluorescent microspheres 24 h after applying them to the cell surface. (Left) Bright-field pictures; (right) corresponding confocal pictures showing the microspheres as red fluorescent dots. Note the progressive displacement of four microspheres from the dome toward the shank of the cell as the cell grows. Bar = 5 μm. (Bottom) Distribution of angles between the cell surface and the growth direction (sectors); (left) red line and tick marks denote the mean and standard deviation. (Right) Angle values plotted as a function of the meridional abscissa |s|, showing that the angle is stable regardless of position in the dome (red line: linear regression). Data are available as Supporting Information S1 Data. S1 Fig illustrates a representative sample of the data.

The prostrate filaments of the alga *Ectocarpus* develop via tip growth [28]. Prior to identifying the underlying mechanisms, a series of additional biophysical information was collected. The cell wall dye calcofluor-white was used in pulse-chase experiments to measure the growing region more precisely. The stain was localized in the first 3 μm distal from the tip of the cell, corresponding to roughly half of the dome (Fig 2A). To assess the direction of growth at the local level, 0.2 μm–diameter fluorescent beads (microspheres) were loaded on the surface of the cell, and their displacement during growth was followed using time-lapse microscopy. This method was initially developed for other plant cell types [30] and recently optimized for *Ectocarpus* [31]. Bead trajectories were drawn, and the angles these trajectories made with the cell contour were calculated. Statistical analyses of the distribution of these angles showed a moderate deviation (relative mean difference < 10%) between the fluorescent marker trajectory and an orthogonal displacement pattern. Moreover, linear regression exhibited no systematic dependence of the angle on meridional abscissa (Pearson correlation coefficient r = −0.03), indicating that growth can be considered orthogonal to the cell surface in the dome independently of the position along the meridional abscissa (Fig 2B and S1 Fig).

### The dome of the *Ectocarpus* apical cell undergoes high wall stress

**Fig 3:**
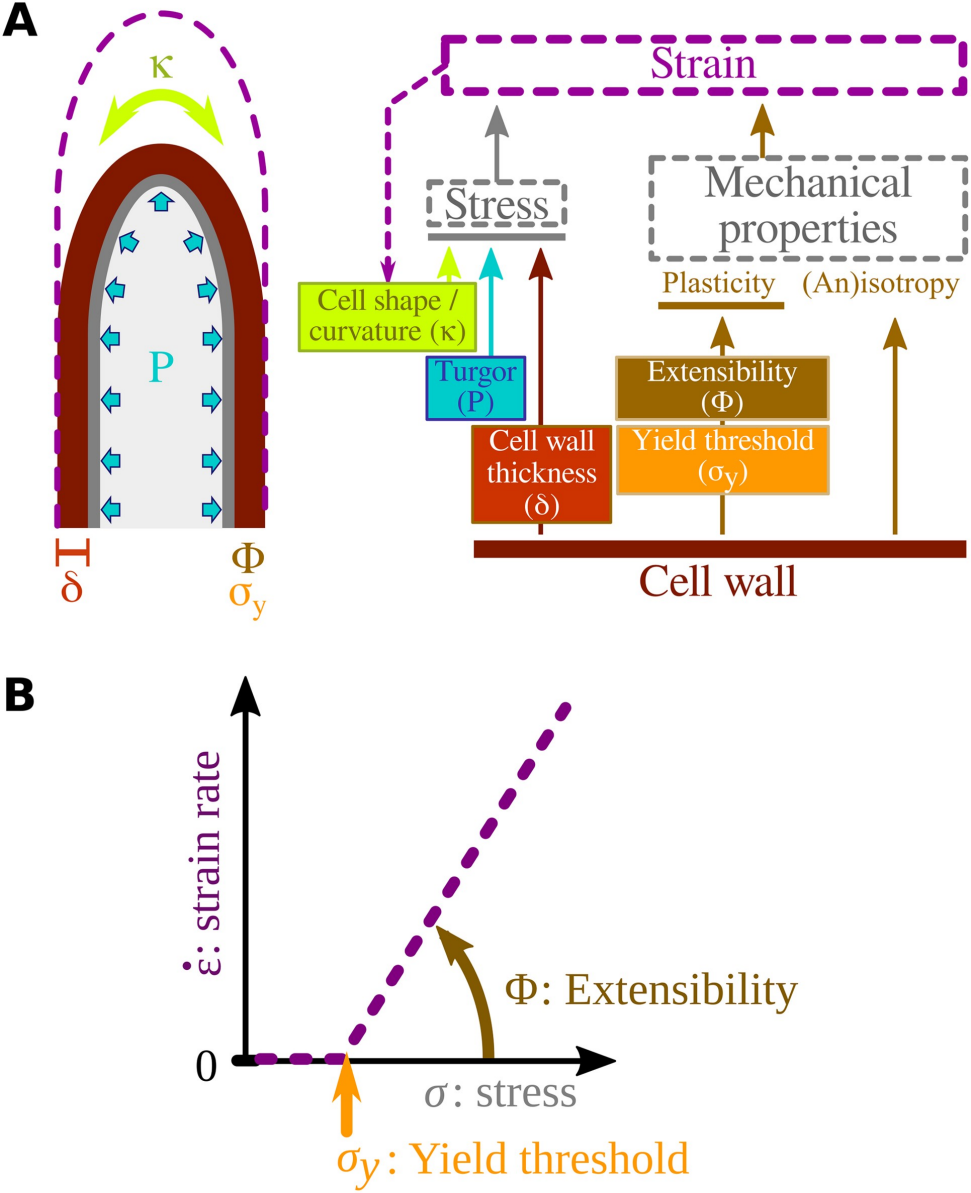
Biophysical model of tip growth. (A) Diagram showing the relationship between the different factors involved in cell wall growth. Wall stress depends on cell turgor (P), cell curvature (κ), and cell wall thickness (δ). In the viscoplastic model [33], the strain rate (purple dashed lines) at each point of the cell surface is a function of wall stress and the mechanical properties of the cell wall (i.e., isotropy and propensity to yield represented by extensibility Ф and the yield threshold σ_y_). Strain results in a new cell shape (dashed arrow). (B) Strain rate as a function of stress, according to the Lockhart law for growth of viscoplastic cell walls.

In plants, turgor is the essential force for growth, whatever the mechanical properties of the wall. Although it exerts the same pressure throughout the cell wall, the resulting wall stress σ_e_ perceived locally in the cell wall varies because of fluctuation in local measurements (see below). The calculation of wall stress is independent of the mechanical features of the cell wall (e.g., elastic, viscoplastic, plastic) and therefore independent of the biophysical model used subsequently. In addition to turgor, wall stress depends on the curvature of cell κ and on the thickness of the cell wall δ at each position of the cell surface (Fig 3A). Wall stress is partitioned into three directions: meridional (*s*), circumferential (*θ*), and normal (*n*) (see equation S2 in S1 Text). Because the cell wall is thin compared to cell size, the normal component of the wall stress is considered negligible compared to the two others [32].

**Fig 4:**
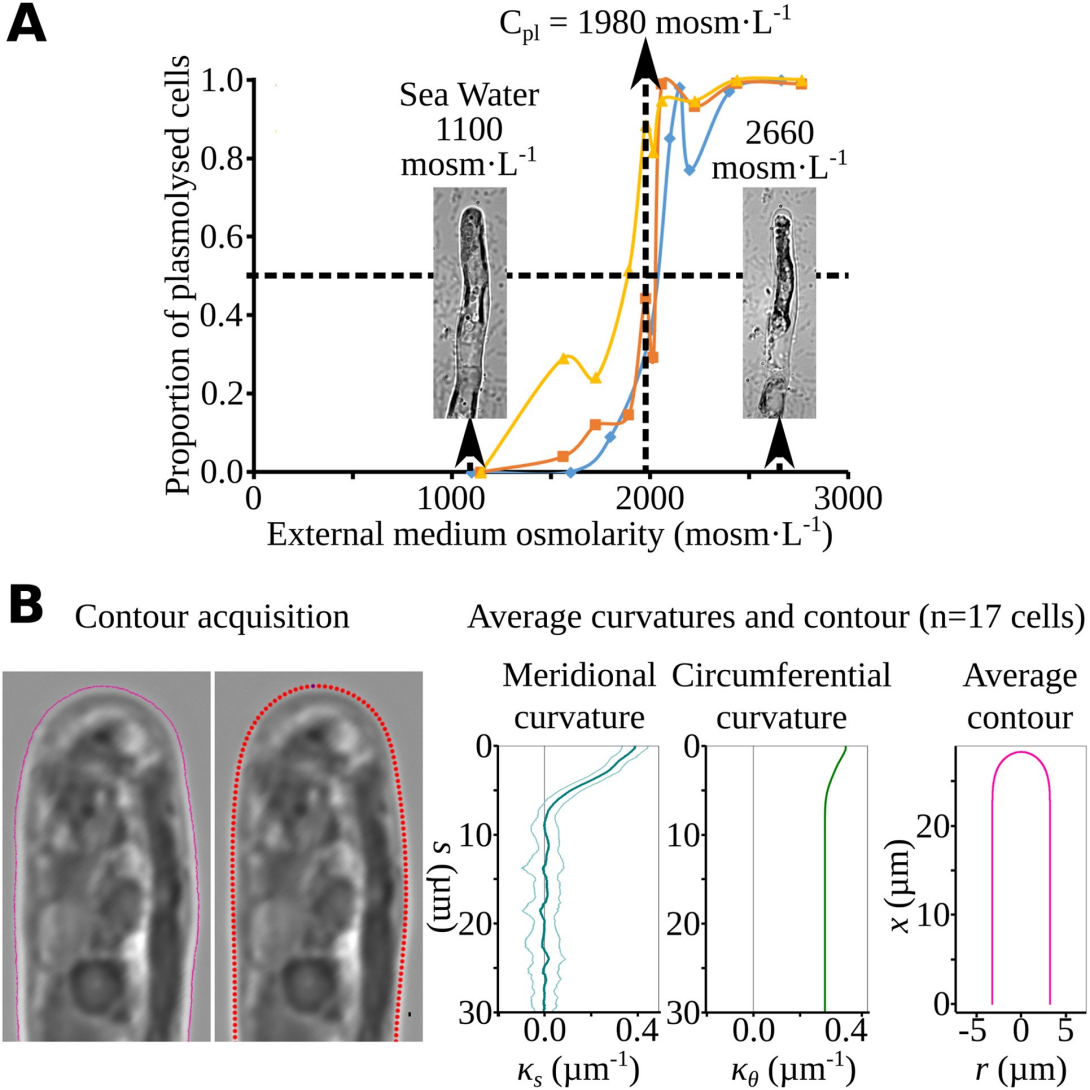
Turgor and curvature of the apical cells. (A) Turgor value in apical cells measured using the limit plasmolysis method [34]. Different osmolarities (C_e_) were applied to *Ectocarpus* filaments, and plasmolysis was monitored in apical cells (*n* > 100 for each osmolarity). Limit plasmolysis concentration (*Cpl*), which is the solute concentration for which 50% of apical cells are plasmolyzed, was 1,980 mOsm L^−1^ (each color represents an independent experiment [*n* = 3]). Corrections, as explained in the Materials and methods section, led to a final turgor value of 0.495 MPa. Data are available as Supporting Information S3 Data. (B) Apical cell curvature. (Left) *Ectocarpus* apical cell contour was drawn manually on microscope images. (Middle) From the contour of each cell, a smoothed cubic spline was computed. (Right) The meridional curvature of each cell was calculated from the discretized contour. All curvature series (for *n* = 17 *Ectocarpus* apical cells, S2 Fig) were averaged (blue curve, SD shown as light blue curves), and the mean curvature was used to create a mean contour. Circumferential curvature (green curve) was then inferred from the mean contour. Gray lines are for curvature = 0. The same procedure was used for 6 tobacco pollen-tube cells.

To calculate the wall stress at many different points in an *Ectocarpus* apical cell, we obtained quantitative data for three components: (1) turgor (P), (2) curvature (κ) of the cell surface, and (3) cell wall thickness (δ). First, turgor in the apical cells was measured using the nonintrusive technique of incipient plasmolysis [34] on >100 cells for each of the 10 solutions of different osmolarities used in the experiment (Fig 4A). The value was subsequently corrected to take into account cell shrinking according to the protocol described in [34] (S2 Data). The calculated apical cell turgor was 0.495 MPa, which is about 5 times the atmospheric pressure and is on the same order of magnitude as tip-growing cells from other eukaryotic groups, including the pollen tube (0.1–0.4 MPa, average at 0.2 MPa; [5]).

The second component of wall stress is the curvature of the cell surface (κ). To measure κ, the contour of *Ectocarpus* apical cells was first drawn manually. Then from 17 individual cell contours (S2 Fig), both the meridional and the circumferential curvatures as well as an average cell contour were calculated (Fig 4B). The same procedure was used for the tobacco pollen-tube contour. Compared with the pollen tube, the *Ectocarpus* apical cell displayed a sharper tip and a higher circumferential curvature on the shanks because of its smaller radius.

**Fig 5:**
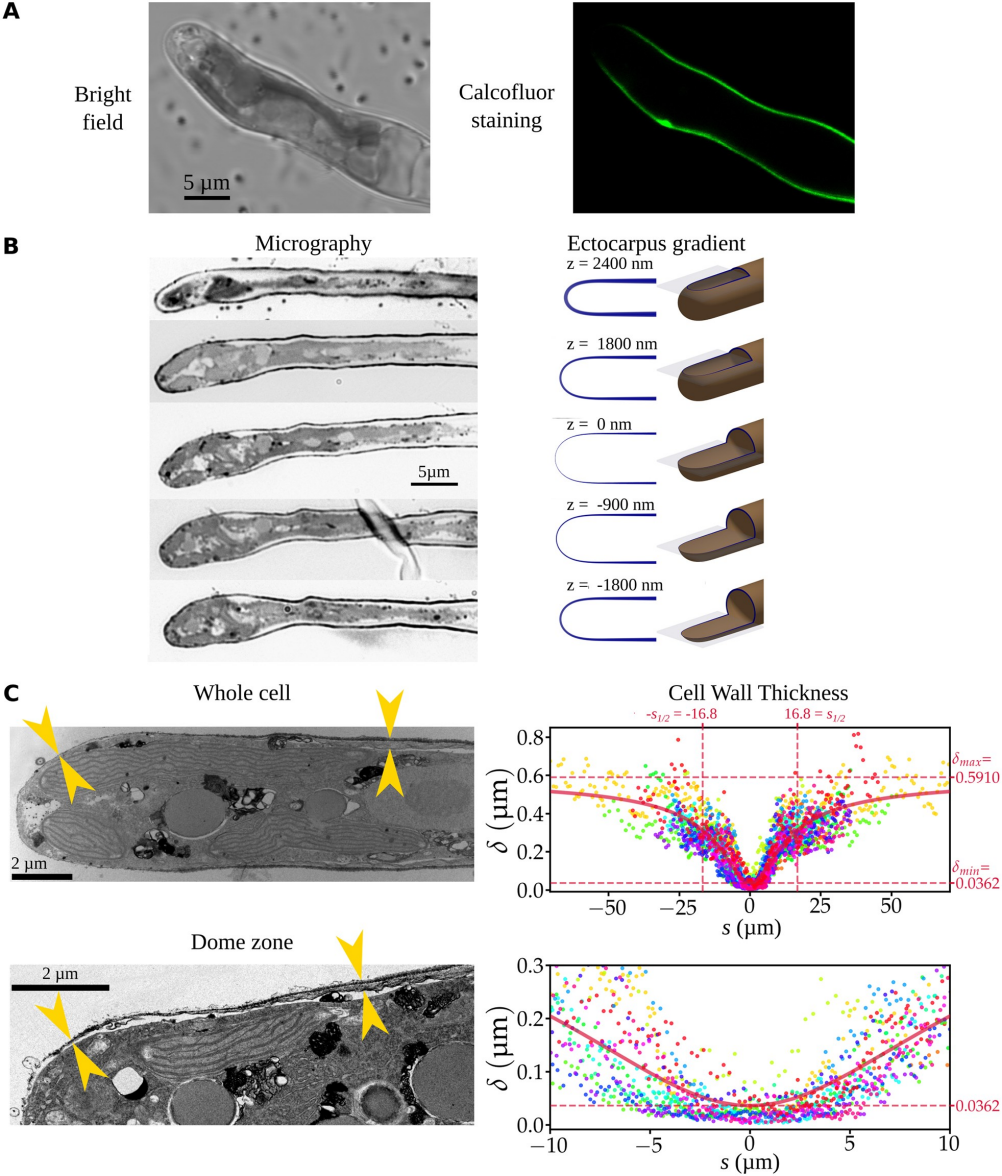
Cell wall thickness of the apical cell. (A) Confocal images of *Ectocarpus* apical cells stained with calcofluor-white. The most apical part of the cell is barely visible because the cell wall is thin. (B) Serial sections (300 nm thick) of an apical cell compared with theoretical sections with the cell wall gradient observed in (C). Theoretical sections were rendered using Persistence Of Vision ray-tracing software [37]. In the meridional position, the cell wall is barely visible at the tip, whereas it is clearly visible in the shanks. In nonmeridional sections, the cell wall is visible both at the tip and in the shanks. (C) Left: Ultrathin (70 nm) longitudinal sections of apical cells observed by TEM, showing the cell wall thickness gradient from the tip to the base of the cell, from a large field view (top) and from a close-up focused on the dome region (bottom). (Right) Plotted distribution of the corrected cell wall thickness values measured every 386 nm in average as a function of the meridional distance (*s*) from the tip (*s* = 0) to *s* = ±70 μm on both sides. Each color corresponds to one cell (*n* = 15 cells); each dot corresponds to one value measured on one given cell. The curve shows the theoretical gradient adjusted to the data, according to a law adapted from Pearson’s function. Adjusted cell wall width at s = 0 is δ = 36.2 nm, and the plateau on the shanks is δ = 591 nm. The distribution in the dome area is shown (bottom). See the whole set of photos in S3 Fig and the whole set of measurements in S4 Data. TEM, transmission electron microscopy.

The third component of wall stress is cell wall thickness (δ). Staining of *Ectocarpus* filaments with calcofluor-white, which labels cellulose (1–4) and callose (1–3)-beta-D-glucans [35], displayed a very clear gradient in thickness from the tip to the shanks of the apical cell (Fig 5A, also visible in 3D reconstruction from confocal microscopy). However, cellulose microfibrils are only a minor component of the brown algal cell wall (8% maximum dry weight), because they are immersed in a more abundant matrix of polysaccharides (45% dry weight) made of alginates (linear polymers of β-[1→ 4]-D-mannuronate and α-[1→ 4]-L-guluronate) and fucans (α-L-fucosyl residues) [23,36]. Therefore, we prepared longitudinal sections of apical cells. First, 300 nm–thick serial sections showed a gradient in thickness increasing from the tip to the shanks in the most meridional sections (Fig 5B, middle section), whereas the thickness appeared even throughout the cell in the most tangential sections (Fig 5B, top and bottom sections). Measurements of cell wall thickness across 70 nm–thick serial sections observed with transmission electron microscopy (TEM) further supported the presence of a cell wall thickness gradient (Fig 5C). Overall, 2,500 measurements were corrected (see Materials and methods) and plotted as a function of *s*. The distribution depicted a gradient that could be modeled as a Pearson-like function characterized by the lowest value δ_min_ = 36.2 nm at the tip (*s* = 0), the asymptotic maximum value δ_max_ = 591 nm, and a midpoint at *s*_*1/2*_ = 16.8 μm (Fig 5C). Cell wall thickness at the distal part of the dome (*s* = 8 μm) was 169 nm, i.e., 4.7 times the thickness at the tip (Fig 5C, close-up).

The establishment of a cell wall thickness gradient contrasts with most tip-growing cells from other eukaryote groups [13,38], in which cell wall thickness is either constant (e.g., 250 nm in pollen tube [39]) or higher at the tip (e.g., oscillating growth in the pollen tube [40,41]).

**Fig 6:**
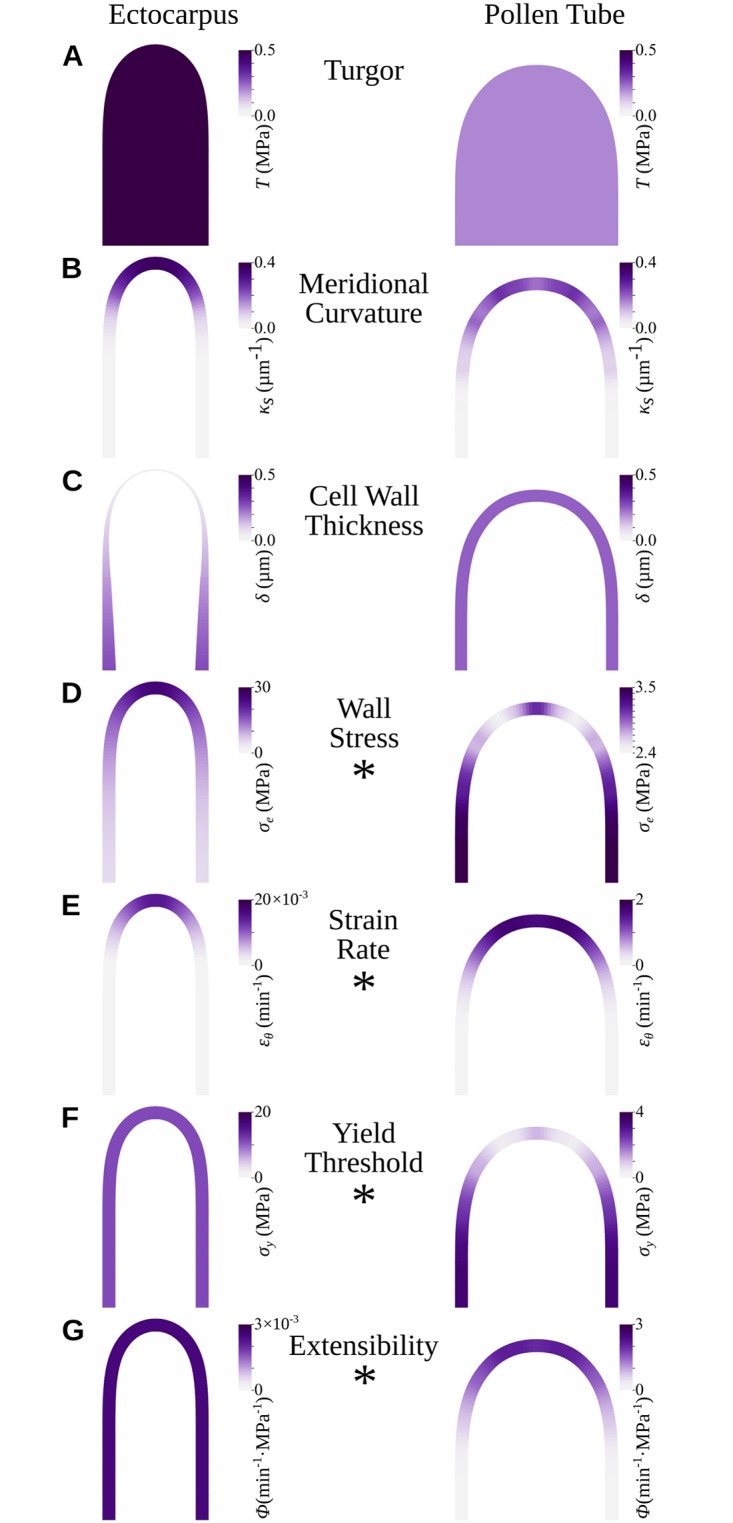
Diagrams summarizing the biophysical properties of two tip-growing cells: *Ectocarpus* filament apical cell and the tobacco pollen tube. 2D profiles are shown. (A) Turgor. (B) Meridional curvature. (C) Cell wall thickness. (D) Wall stress. (E) Strain rate pattern. (F) Cell wall plastic yield threshold. (G) Cell wall plastic extensibility. Note that the color scale differs between *Ectocarpus* and pollen tube in (D–G), indicated with an asterisk (*).

Fig 6A, 6B and 6C show a diagram of these biophysical factors in both *Ectocarpus* and pollen tube apices.

**Fig 7:**
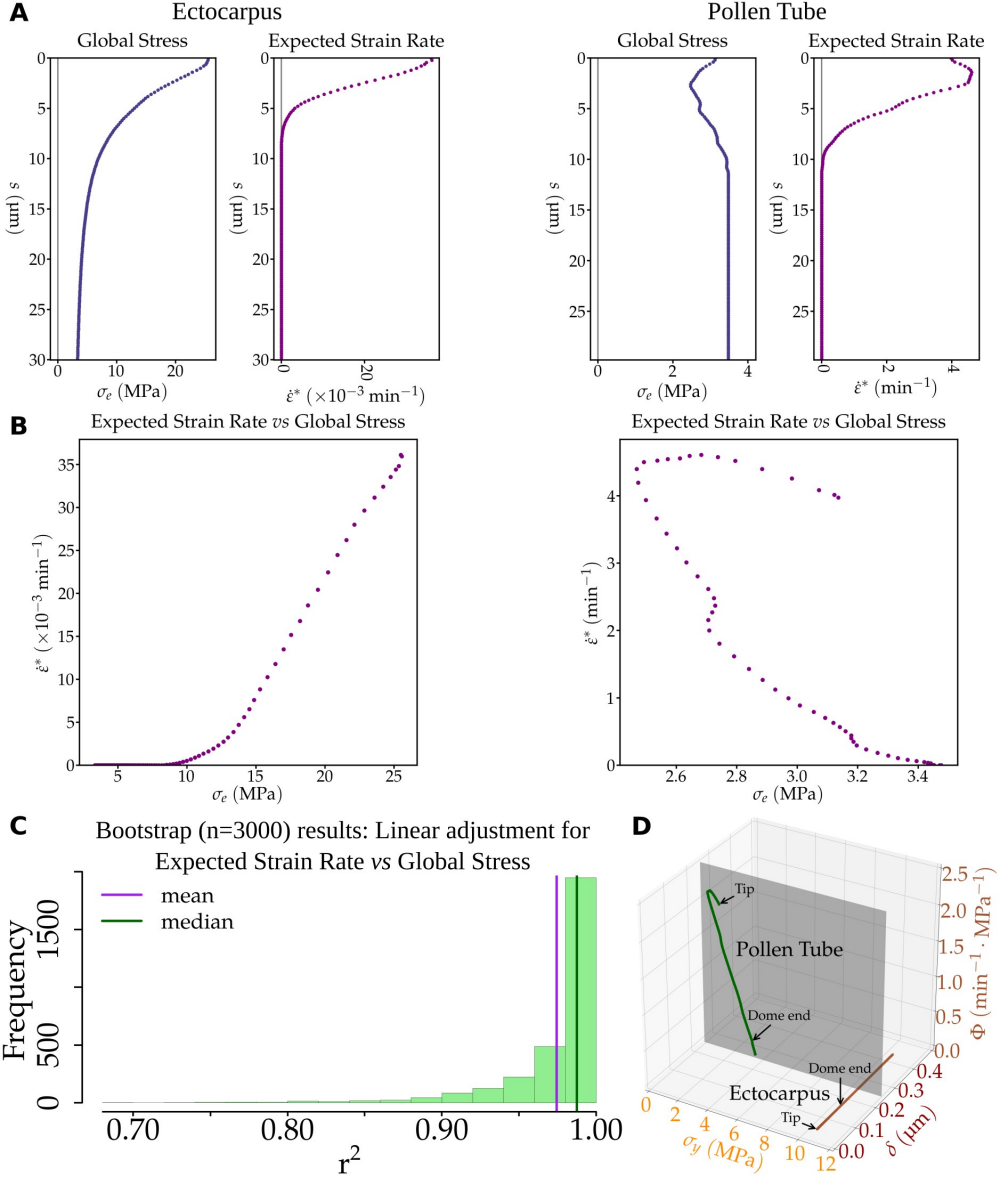
Contribution of the cell wall biophysical factors in *Ectocarpus* and pollen-tube tip growth. (A) For each cell type, the global stress σ_e_ was computed using measured values of turgor, curvature, and cell wall thickness (equation S2 in S1 Text). Knowing normal velocity *V*_*n*_ at each point, the expected strain rate ${\dot{\varepsilon }}^{*}$ was computed according to equation S10 in S1 Text. Note the different scales between *Ectocarpus* and the pollen tube. (B) Relationship between stress and expected strain rate in *Ectocarpus* apical cells (left) and in the tobacco pollen tube (right): instead of plotting each value against *s*, these values were plotted against each other to show how the stress results in strain. In *Ectocarpus*, but not in the pollen tube, ${\dot{\varepsilon }}^{*}$ behaves according to the Lockhart equation $\dot{\varepsilon }=\Phi (\sigma_{e}-\sigma_{y})if\sigma_{e}>\sigma_{y};\dot{\varepsilon }=0$ otherwise, with constant values for Φ and σ_*y*_ (compare with Fig 3B). (C) Robustness of this result was tested using a bootstrap analysis with 3,000 replicates. For each sample, the linearity of the part of the curve (where σ_e_ > σ_y_) was estimated by linear regression. The distribution of the values of r^2^ shows that linearity is well supported (see also S4 Fig). Data are available as S4 Data. (D) Relationship between the three biophysical features of the cell wall: plastic yield threshold (*σ*_*y*_, x-axis), thickness (δ, y-axis), and plastic extensibility (Φ, z-axis). In *Ectocarpus*, only variation of δ accounts for tip growth (brown line), whereas in pollen tubes, both *σ*_*y*_ and Φ vary while the wall thickness remains constant (green line).

Using this set of biological data, wall stress was calculated in both the meridional (σ_s_) and the circumferential (σ_θ_) directions, which ultimately allowed calculating the overall wall stress σ_e_ (Fig 3A; equation S3). Although σ_e_ fluctuates between 2.5 and 3.5 MPa (with the lowest value in the dome) in the pollen tube, it reaches a maximum of 25.6 MPa in the *Ectocarpus* tip and decreases distally, reaching values similar to that in the pollen tube 70 μm away from the tip (Figs 6D and 7A). This stress value in the dome of *Ectocarpus* apical cells is remarkably high compared to other tip-growing cells, which, moreover, show the opposite stress gradient, increasing from tip to shank.

### Spatial variation in wall stress accounts for the viscoplastic strain pattern in *Ectocarpus*

#### Implementation of the viscoplastic model

We investigated how the wall stress gradient observed in *Ectocarpus* apical cell impacts the growth rate and geometry. Although plant cell wall growth used to be attributed to plastic deformation only [42], some biophysical models consider the intrinsic elasticity of the cell wall as a significant factor in cells subject to transpiration or water stress [43]. However, because *Ectocarpus* growth is extremely slow (approximately 300 times slower than pollen tubes) and takes place always in immersed conditions (in lab), we considered that the elastic component due to rapid and reversible fluctuations of osmotic pressure is negligible in the *Ectocarpus* apical cell and that the growth process relies only on the plastic component of the cell wall.

The physical laws governing plastic growth of a cell subject to turgor pressure were initially established by Lockhart [44]. The same scheme is applied here at the scale of an infinitesimal piece of cell wall, which is distorted if the stress exceeds the yield threshold (σ_e_(s) > σ_y_(s), see Fig 3B [45]). Wherever this condition is met, local deformation occurs with an orientation and rate (strain rate $\dot{\varepsilon }(s)$) that depend on the local cell wall plastic extensibility Φ(s) and intrinsic anisotropy (Fig 3A) [33] (equation S5 and details in S1 Text).

Therefore, to obtain data on the structural anisotropy of the cell wall in *Ectocarpus* apical cell, we denatured the cell wall and observed the remaining cellulose fibers using AFM. Apparent diameter of cellulose microfibrils is in agreement with previously published results (12.6 ± 4.9 nm) [46]. Images showed that cellulose microfibrils were intermingled throughout the cell surface in the dome, indicating that the main, stiffer components of the cell wall have no specific direction in this dimension (S5A Fig, left, middle). Similar organization was observed in partially denatured cell walls, showing that the treatment does not displace the cellulose microfibrils (S5A Fig, right). This finding demonstrates that the cell wall of the tip is transversely isotropic, a feature shared by other *Ectocarpus* cell types [46].

#### *Ectocarpus* tip growth mirrors the canonical Lockhart curve

In addition to wall stress, cell wall isotropy, and the direction of deformation as assessed above, the strain rate depends largely on the cell wall mechanical properties. It is currently impossible to gain direct experimental access to the values of yield stress threshold σ_*y*_(*s*) and extensibility Φ(*s*) at every position in the cell wall during growth. Although AFM nano-indentation allows inferring intrinsic cell wall mechanical properties such as elasticity (elastic modulus), adhesion, and potentially plasticity in the z-axis, it does not account for forces in the x- and y-axes at play during growth [47]. Nevertheless, transverse isotropy of the cell wall and orthogonal growth together make equations of the viscoplastic model tractable. Therefore, the expected strain rate $\dot{\varepsilon }*(s)$ can be expressed as a function of local geometrical and physical values and without any prior knowledge of Φ(*s*) and σ_*y*_(*s*) (see details in S1 Text). Briefly, the local velocity, normal to the cell surface (*V*_*n*_), is deduced from self-similar growth [48] (i.e., growth without distortion, globally similar to axial translation). Then, the rearrangement of the physical relationships leads to:

$${\dot{\varepsilon }}^{*}=\frac{K\kappa_{\theta }}{\sigma_{\theta }-\nu \sigma_{s}}V_{n},$$

where all terms are functions of *s*, which was omitted for readability: *κ*_*θ*_ the circumferential curvature; *σ*_*s*_ and *σ*_*θ*_ the meridional and circumferential components of stress, respectively; *ν* the flow coupling; and *K* a normalization factor. With their dome shape, *Ectocarpus* and the tobacco pollen tube are expected to show similar patterns of stain rate (Figs 6E and 7A). We observed a much lower rate for the brown alga because of its slower growth rate (2.5 μm h^−1^ compared to 540 μm h^−1^ for the pollen tube).

Then, we examined the two position-dependent factors: the stress and the expected strain rate. We plotted the expected strain rate as a function of wall stress along the cell: ${\dot{\varepsilon }}^{*}(s)=f(\sigma_{e}(s))$ (Fig 7B). The plasticity values Φ and σ_y_ were not used to plot this curve. However, for each position s, the viscoplastic strain rate resulted from the local stress according to the Lockhart equation for the viscoplastic strain rate:

$$\dot{\varepsilon }(s)=\Phi (s)(\sigma_{e}(s)-\sigma_{y}(s)).$$

Therefore, in the particular case when Φ and σ_y_ do not vary with s, the plot for ${\dot{\varepsilon }}^{*}(s)=f(\sigma_{e}(s))$ is expected to display the characteristic shape of a Lockhart curve (Fig 3B). This was observed for *Ectocarpus* (Fig 7B left) but not for the pollen tube (Fig 7B right). We inferred from this result that, in contrast to the pollen tube, Φ and σ_y_ both must remain constant throughout the apical cell in *Ectocarpus*. Instead of interpreting Fig 7B as a result of constant Φ and σ_y_, it remains mathematically possible to imagine that σ_*y*_ and/or Φ change in the cell so that a decrease of one compensates the simultaneous decrease of the other. At the cellular level, this compensation would result for example in softening the wall by lowering σ_y_ and simultaneously hardening it by lowering Φ. However, this combination of variations is not parsimonious and is counterintuitive. Furthermore, it is incompatible with experimental data obtained from plant cell walls in which simultaneous variations of Φ and σ_y_ were always opposing, such that they modify the cell wall stiffness in the same direction [49], in agreement with physicochemical cell wall models (e.g., [50]). This is what is observed for the pollen tube (Fig 7B, right) and is widely supported by experimental evidence [51].

To test the robustness of the strain-stress analysis in *Ectocarpus*, we conducted a bootstrap assay using 3,000 resampling sets among the cells used to compute the average contour and those used to infer the cell wall thickness gradient. As a test to assess the similarity of the curve ${\dot{\varepsilon }}^{*}=f(\sigma_{e})$ with the Lockhart function, we verified the linearity of the increasing part of the curve (i.e., for points having σ_e_ > σ_y_,). The mean linear regression r^2^ value was 0.974, and for 95% of the samples r^2^ was higher than or equal to 0.907 (Fig 7C). Thus, despite variation in cell shape and cell wall thickness between samples, the fit with the Lockhart curve remained very robust (see also S4 Fig).

#### Inferred viscoplastic features of *Ectocarpus* apical cell wall and effect of auxin

**Fig 8:**
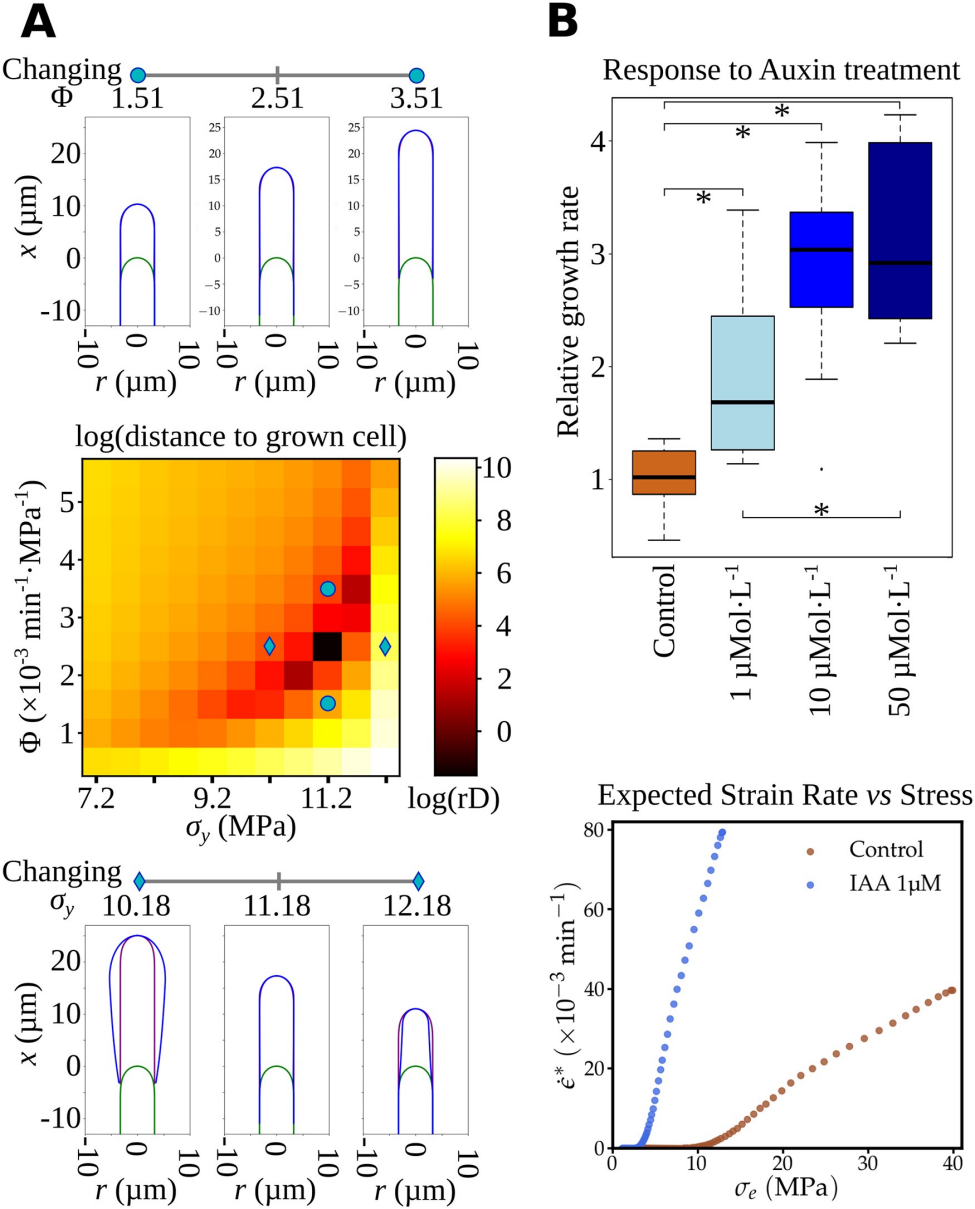
Impact of yield threshold (σ_*y*_) and extensibility (Φ) variations on *Ectocarpus* tip growth. (A) Simulation of tip growth in *Ectocarpus* with varying extensibility (Φ) and yield threshold (σ_y_). (Middle) Heatmap representing the logarithm of mean weighted distance residuals (rD) for a range of σ_y_ (horizontal axis) and Ф (vertical axis) (one complete simulation for each pair of σ_y_ and Ф values). The darker the color, the lower the rD and the better the simulation. rD was calculated as the linear distance of points sharing the same meridional (*s*) distance between the simulated final cell contour and the initial one translated forwardly of 25 μm. Optimized values were 2.51 MPa^−1^ for the cell wall extensibility (Ф) and 11.18 MPa for the yield threshold (σ_y_). See also S1 Movie showing the time course of the 2D simulation. (Bottom) Impact of variation of cell wall yield threshold σ_y_ on tip-growth simulation. The diagram shows the 2D profile of apical cells before the simulation (initial stage, green contour) and at the end of the simulation (blue contour). The purple contour represents the translated initial shape to help comparison with the initial contour. σ_y_ values were 10.18, 11.18, and 12.18 MPa (diamonds on the heatmap). Simulations were run for 5 h 27 min, corresponding to a growth of 25 μm forward for the fastest simulation. See S2 Movie for time course of the 2D simulation with varying σ_y_. (Top) Impact of the cell wall extensibility Ф on tip-growth simulation (same color code as in the bottom figure). Ф values were 1.51, 2.51, and 3.51 × 10^−3^ min^−1^ MPa^−1^ (circles on the heatmap). Simulations ran until the first simulation reached 25 μm in distance. See S3 Movie for time course of the 2D simulation with varying Ф. (B) Response to auxin treatment. (Top) The linear growth rate (ΔL/Δt) was measured 24 h after adding 1, 10, or 50 μM of IAA. Relative growth rate was calculated as the ratio to the mean growth rate in the control condition (2 μM NaOH, see Materials and methods for details). * denotes pairs of conditions for which a pairwise Mann-Whitney tests showed significant differences (*p*-value < 0.05 after Holm correction for multiple tests). (Bottom) Expected strain rate versus stress for control conditions and in the presence of 1 μ Mol L^−1^ IAA. The curve shows that both σ_y_ and Ф are affected by the presence of IAA: σ_y_ decreases while Ф increases, both modifications corresponding to a cell wall–loosening effect. Data are available as S6 Data. IAA, indole-3-acetic acid.

The previous section showed that in the context of the viscoplastic model, Φ and σ_*y*_ values must be constant throughout the cell. The values for these two biomechanical parameters are so far unknown, but they can be inferred from long-term simulations of tip growth in *Ectocarpus*. Simulations were run for 600 steps of approximately 40 nm of linear progression each, over a distance of 25 μm corresponding to about 5 times the dome length. They showed that the constant values σ_*y*_ = 11.18 MPa and Φ = 2.51 × 10^−3^ MPa^−1^ min^−1^ made it possible to maintain the apical cell shape during growth (Fig 8A, middle; S1 Movie). Simulations with different pairs of cell wall Φ and σ_y_ values did not result in the expected self-similar growth and instead produced either misshapen cells when varying σ_y_ (Fig 8A, bottom; S2 Movie) or inappropriate growth rates when varying Φ (Fig 8A, top; S3 Movie).

To test the model experimentally, we treated the apical cells with 3 concentrations of auxin indole-3-acetic acid (IAA). This phytohormone, present in *Ectocarpus* filaments [29], sped up linear tip growth (Fig 8B, top) and reduced turgor in the apical cell (0.186 MPa instead of 0.495 MPa in the control, S7 Data), but no modification of the original cell shape could be noticed. Using these biophysical measurements, and assuming that the thickness gradient was not modified during this experimental time lapse, we managed to simulate tip growth again with constant values of plastic extensibility and yield threshold along the cell, similarly as in the control conditions. In addition, although constant along the cell, Φ and σ_y_ values were different from those in the control: in response to 1 μM IAA, Φ increased to 13.35 × 10⁻³ min⁻¹ MPa^−1^ (i.e., 5.3 times higher than in the control), and σ_y_ decreased to 4.20 MPa (2.7 times lower) (Fig 8B bottom). A similar response has been reported in land plants: tip growth increases in IAA-treated pollen tubes. Biophysical measurements show that IAA-treated hypocotyls of *Vigna* display a higher strain rate correlated with an increased Φ and a decreased σ_y_ [49]. Therefore, notwithstanding the phylogenetic distance between the two eukaryotic phyla, auxin may have the same effect on cell wall mechanical properties: facilitation of the plastic deformation to increase growth rate. In addition to this hypothesis, these data support the model in which Φ and σ_y_ remain constant along the cell.

### The cell wall thickness gradient affects both cell shape and growth rate

Using the model, we tested the impact of the cell wall thickness gradient on tip shapes and growth rates. Steeper or gentler cell wall thickness gradients were sufficient to substantially alter the typical *Ectocarpus* cell shape and growth rate, suggesting that the cell wall thickness gradient must be tightly regulated in vivo (S6 Fig, central column; S4 Movie). However, cells display some significant variation in cell wall thickness (Fig 5C). In vivo observation of *Ectocarpus* tip growth also showed variability in growth rate and in cell shape (e.g., displayed in S2 Fig), which may be due to transitory variation in cell wall thickness. The extremely low growth rate of this species can easily allow the activation of regulatory mechanisms that adjust the cell wall thickness gradient through modifications in cell wall biosynthesis.

We performed the same experiments on cells with three different initial cell shapes (flat, typical *Ectocarpus*-like, and sharp). Using the *Ectocarpus* cell wall thickness gradient “Normal” resulted in convergence of the resulting shapes to the typical *Ectocarpus* shape (S6 Fig, middle row; S5 Movie). Therefore, the cell wall thickness gradient may also govern the tip resilience to deformation so that initial cell shape can be recovered after transient deformation (e.g., due to an accident during growth). When simulations used a modified cell wall thickness gradient (“Steep” or “Gentle”) on these different initial cell shapes, all cells grew and converged to the same final shape specific to the given thickness gradient (S6 Fig, top and bottom rows; S6 Movie). These simulations supplement those by Dumais and colleagues [33], who explored various gradients in Φ and σ_*y*_ in a context where cell wall thickness was constant.

### Maintenance of the cell wall thickness gradient

**Fig 9:**
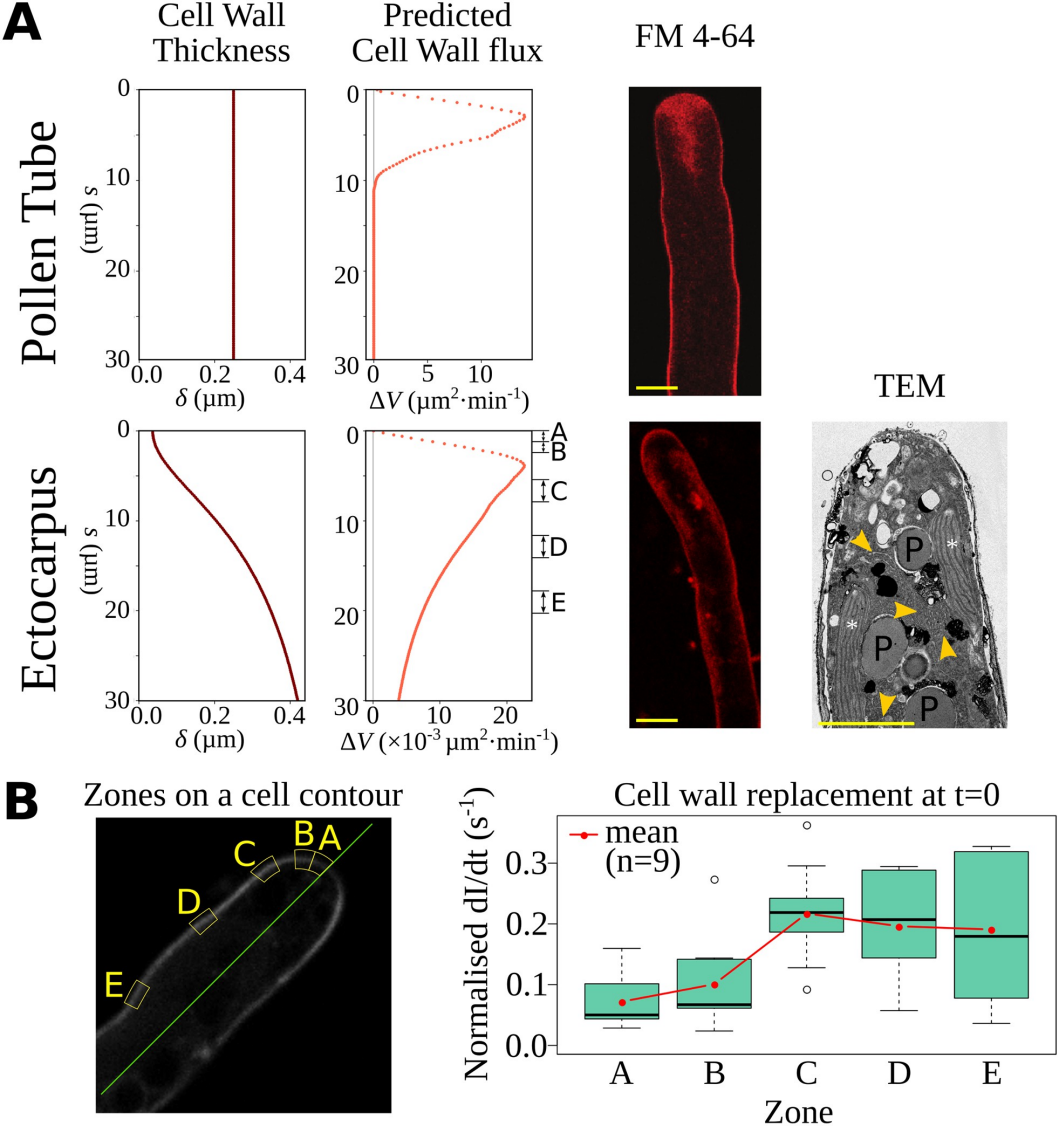
Impact of the cell wall thickness gradient and pattern of cell wall biosynthesis. (A) Dynamics of cell wall synthesis in the pollen tube (top) and *Ectocarpus* apical cell (bottom). From left to right: cell wall thickness δ from which the computed cell wall flux was inferred using the model. Note the different x-scales between *Ectocarpus* and the pollen tube. Vesicle pattern displayed by FM4-64 labeling. Confocal image about 30 min after addition of FM4-64 at RT in living *Ectocarpus* and in the pollen tube (Courtesy of G. Grebnev and B. Kost, Erlangen Univ., Germany). Bar = 5 μm. Longitudinal sections observed using TEM. No specific network of vesicles was observed in the dome of the *Ectocarpus* apical cell (see [55] for a comparative image of the pollen tube). Instead, chloroplasts and associated reticulum (see [14] for the description of the overall intracellular organization) are present all along the cell axis. White stars: chloroplasts; orange arrow heads: CER; P: pyrenoids. Bar = 5 μm. (B) FRAP experiment. (Left) Definition of the zones A–E from which fluorescence recovery was measured (also shown in panel A). (Right) Quantification of cell wall replacement expressed as the increase in normalized fluorescence intensity at t = 0 (time of photobleaching). See S7 Fig for details about the FRAP analysis. Data are available as S8 Data. CER, chloroplastic endoplasmic reticulum; FRAP, fluorescence recovery after photobleaching; RT, room temperature; TEM, transmission electron microscopy.

The preponderant role of the cell wall thickness gradient in the control of tip growth raises the question of how this gradient is established and maintained. Calculations considering the cell wall extension rate and the maintenance of the cell wall thickness gradient during growth allowed inference of the level of cell wall material delivery and/or biosynthesis along the cell. According to this calculation, the overall delivery rate of cell wall material and/or synthesis in the pollen tube is much higher than in *Ectocarpus* (note the different scales of the x-axis in Fig 9A, left, top versus bottom). The maximum culminates 3.0 μm away from the most distal position and drops to nil in the tube shanks (Fig 9A, top left). This calculation is in agreement with former in situ observations using FM4-64 that labels both endocytic and exocytic vesicles [52–54] (Fig 9A, top middle). This pattern is also in agreement with TEM observations in pollen tubes [55] and in other tip-growing walled cells where vesicle trafficking is concentrated in the most distal part of the tip (root hairs and green algae reviewed in [56], ascomycetes [13]).

This mechanism contrasts with *Ectocarpus*, in which the cell wall flux is predicted to be significant in the shanks of the cell (Fig 9A, bottom left). How the cell wall is constructed in brown algae is still largely unknown. Cellulose may be synthesized from cytosolic uridine diphosphate (UDP) glucose via linear complexes of cellulose synthases localized in the plasma membrane, where they elongate cellulose microfibrils into the cell wall [57]. It is still unknown how the other main cell wall components (alginates and fucans) reach the cell wall at the tip of the *Ectocarpus* apical cell, but the current delivery mode is thought to be through Golgi-derived vesicles [58]. Therefore, we used FM4-64 to investigate the pattern of vesicle trafficking in *Ectocarpus*. FM4-64 displayed an homogeneous spatial pattern all along the cell, with no specific vesicle localization (Fig 9A, bottom middle). TEM observations provide further support because vesicles were never concentrated in any of the meridional sections of the dome of an apical cell (bottom right). Instead, chloroplasts and chloroplastic endoplasmic reticulum (CER) involved in the production of photosynthates [14] were observed in the dome as well as in the shanks of the cell (Fig 9A, bottom right). Therefore, observations of biological activity are compatible with the establishment and maintenance of a cell wall thickness gradient at an extremely slow rate, where CER can deliver the main components of the cell wall all throughout the cell with nevertheless the highest rate in the dome. To confirm this initial observation, we performed fluorescence recovery after photobleaching (FRAP) assays on *Ectocarpus* apical cells. We compared the fluorescence signal recovery dynamics in 5 different zones in the dome and shanks of the cell (Fig 9B, left). Considering the increase in the fluorescence signal over time, we used the normalized slope at *t* = 0 as a proxy for the intensity of membrane replacement by exocytosis, potentially reflecting cell wall–building activity (S7 Fig). The results showed that the exocytosis rate (Fig 9B right) reflects the cell wall flux inferred from the model (Fig 9A, bottom left). Our FRAP experiments support the prediction of the highest exocytosis activity at the base of the dome (zone C at s ≈ 5 μm) and significant activity in the shanks (zone E ≈ 10 μm from the dome end). Altogether, FRAP and TEM observations are compatible with the calculation of the cell wall flux inferred from the model.

## Discussion

Using a combination of serial longitudinal sections observed by TEM and optical microscopy, we first showed that *Ectocarpus* displays a gradient of cell wall thickness in its apical cells. Reports indicate that cells from other organisms display a cell wall thickness gradient. However, in most cases, accurate measurements could not be obtained from the methods employed such as epifluorescence microscopy of plant trichomes stained with propidium iodide [59] or bright-field microscopy of entire ghost cell walls of healing tips of the green alga *Acetabularia* [60]). Recently, Davì and colleagues [61] developed a technique on fission yeast enabling a resolution of 30 nm in living cells. However, this resolution is in the lower limit of *Ectocarpus* cell wall thickness, and TEM therefore appeared to be the most reliable technique. The accuracy of the TEM technique revealed a very steep thickness gradient ranging from 36 nm at the very tip to 169 nm at the base of the dome, corresponding to an average slope of 1.6%. No such gradient has been reported in the growth zone of other organisms. In the diffusely growing trichome of *Arabidopsis*, the cell wall thickness increases in the cell with a slope of 0.3% [59]. In the apical cell of *Neurospora*, cell wall thickness is constant in the dome and gradually increases along the shanks [62], a pattern similar to that observed in fission yeast [61].

In terms of biophysics, this gradient in cell wall thickness resulted de facto in a decrease in the stress from the shanks to the tip. The biological measurements specific to the *Ectocarpus* apical cell (turgor, dome geometry, and cell wall thickness) were integrated in the viscoplastic model initially proposed by Lockhart and further developed for tip growth by Dumais and colleagues [33]. The observed cell wall thickness gradient quantitatively compensated for the reduction of stress with an increase in curvature from the shanks to the tip. After adjusting the plasticity values, the model was able to achieve self-similar growth at the speed observed in vivo. Regarding the cell wall mechanical properties, the model inferred two main differences with the pollen tube. First, the extensibility Φ and the yield threshold σ_*y*_ remained constant throughout the *Ectocarpus* cell, in contrast to the pollen tube models in which the constant thickness of the cell wall necessarily requires modification of the cell wall mechanical properties to allow growth [63]. Plotting σ_*y*_ and Φ together with cell wall thickness δ clearly illustrates the different strategies developed by *Ectocarpus* and the pollen tube (Fig 7D): in *Ectocarpus*, δ is the only varying factor, but in the pollen tube, both σ_y_ and Φ vary, whereas δ remains constant. Using a Lab-on-a-Chip platform, Shamsudhin and colleagues [64] confirmed that the pollen tube displays an apparently increasing elastic modulus from the tip to the shanks, which is correlated with the presence of methyl-esterified pectins [65].

Secondly, compared with the pollen tube, the overall value of strain rate is approximately 100 times lower, but stress is approximately 10 times higher in *Ectocarpus* (Fig 6), suggesting that the *Ectocarpus* cell wall is generally more resilient to yielding during growth. Experimental work is clearly still needed to refine the values of the yield threshold and extensibility, but our calculation of wall stress offers a solid basis on which their order of magnitude can be inferred. Nano-indentation of *Ectocarpus* cell wall produced values of elastic modulus much lower (approximately 1–4 MPa, [27]) than those reported in the pollen tube (approximately 20–400 MPa, [64]). However, the different nano-indentation experimental procedures used in these studies (depth of indentation, shape of the indenter, osmotic conditions, physical model) make comparisons questionable [47]. Nevertheless, the elastic modulus assessed using nano-indentation and the cell wall mechanical properties inferred from the growth model together suggest that *Ectocarpus* is more elastic but less prone to expansion during growth than the pollen tube. The distinction between cell wall elasticity and growth has already been made in the green alga *Chara* [66] and has since been reported in other plant cells (reviewed in [67]). The inverse relationship observed in *Ectocarpus* is fairly compatible with the dual role of the cell wall in brown algae, i.e., coping with frequent environmental changes in osmotic pressure (tides), which requires a high elasticity, and resistance to yielding in the face of high wall stress due to the thin cell wall and high turgor. The lack of a functional relationship between intrinsic elasticity and cell wall extensibility has already been reported in land plants [67]. Likewise, the presence of stiff or soft polysaccharides—as assessed in vitro—does not correlate with the expansion of the cell wall during plant growth (e.g., [68,69] and reviewed extensively in [67]), nor apparently during growth in brown algae [70].

Another puzzling question is how *Ectocarpus* controls the cell wall thickness gradient necessary to ensure the maintenance of cell shape. It is unknown whether cell wall thickness fluctuates during growth, as recently reported in the fission yeast [61], but this fluctuation may account for the variation in cell shape and growth rate observed in living organisms. Nevertheless, the gradient in thickness requires regulation of cell wall biosynthesis, which in brown algae like in land plants involves both in muro cellulose synthesis and the delivery of other components (fucans and alginates in brown algae) through vesicle trafficking (Golgi and flat cisternae respectively in Fucales; [58]). FRAP data showed that the highest exocytosis activity was localized in the basal region of the dome, just before the cell adopts its cylindrical shape. This coincides with the highest cell wall flux computed from the model and with the pattern described in the pollen tube [71,72]. How exocytosis vesicles are targeted to these positions is unknown. In yeast and land plants, mechanosensors localized in muro control cell wall biosynthesis enzymes to modulate cell wall thickness and respond to cell wall damage [60,73]. The *Ectocarpus* genome codes for several mechanosensor proteins (integrins, transmembrane proteins containing the WSC carbohydrate-binding domain [25,74]), and these proteins may well be key regulatory factors in this process.

The palette of tip-growing strategies among species is not restricted to the control of cell wall thickness and of cell wall mechanical properties through pectin methyl-esterification. Other molecular mechanisms, including pectate distortion cycle in *Chara* [75], secretion of glucanases and chitinases in fungi [13], and intussusception in prokaryotes [76], have been proposed to account for the differential cell wall mechanics along the cell. Therefore, distinct key cell wall biophysical factors, and potentially a combination of them [61], appear to have been selected during evolution to achieve cell wall growth. The evolutionary history of brown algae is short (approximately 250 My) and distinct from that of land plants. The marine environment characterized by high physical pressure and ionic concentrations, low gravitational forces, and high drag forces due to tremendous sea currents may have promoted the development of specific, singular strategies in these peculiar organisms. In the case of tip growth, although we cannot formally exclude the possibility that a gradient of wall mechanical properties exists and contributes to morphogenesis in *Ectocarpus*, our results suggest that this organism has favored a singular approach based on cell wall thickness and hence on control of wall stress. The question remains whether the particular features of this organism, including its slow growth, make the control of cell wall thickness more efficient than the control of cell wall mechanical properties.

## Materials and methods

### Culture of *Ectocarpus* parthenosporophytes

Parthenosporophyte filaments of *Ectocarpus* sp. (CCAP accession 1310⁄4) were routinely cultivated in natural seawater (NSW) as described in [77]. For microscopic observations and time-lapse experiments, early parthenosporophytes were obtained from gamete germination on sterile coverslips or glass-bottomed petri dishes.

### Auxin treatments

*Ectocarpus* prostrate filaments were treated with 1, 10, and 50 μM IAA (Sigma-Aldrich I3750) prepared in 2, 20, and 100 μM NaOH, respectively (final concentration). Growth rates were measured for each concentration 24 h post treatment (*n* = 10), using NSW supplemented with 2 μM NaOH as a control. Turgor was measured in 1 μM IAA using 2 μM NaOH as the control (see Measurement of turgor in the apical cell and correction for details).

### Measurement of turgor in the apical cell and correction

*Ectocarpus* filaments were immersed for 1 min in a range of sucrose concentrations (diluted in NSW), and the proportion of plasmolyzed apical cells was measured by counting apical cells (*n* > 100) under an optical microscope. The rate of plasmolysis was plotted against external osmolarity (*c*_*e*_). The limit plasmolysis (*c*_*pl*_) corresponds to the value of *c*_*e*_ at which 50% of apical cells were plasmolyzed. The mean *c*_*pl*_ value was calculated from 3 independent experiments. Solution osmolarities were measured with an osmometer (Osmometer Automatic, Löser, Germany). Because the cell wall of *Ectocarpus* is partly elastic, plasmolyzed cells have a reduced volume that must be taken into account to calculate the real internal osmolarity (*c*_*i*_) and thus the real internal turgor (*P*). To do so, the coefficient of apical cell volume shrinking (*x*, equal to the ratio of the cell volume upon plasmolysis to the cell volume in normal growth conditions) was measured on apical cells (*n* = 9), and the corrected internal osmolarity was calculated as *c*_*i*_ = *x*. *C*_*pl*_. The difference between internal and external osmolarities is *Δc* = *c*_*i*_ − 1,100 with the seawater osmolarity = 1,100 mOsm L^−1^, and the turgor is $P=\frac{c_{i}–c_{e}}{410},$ in MPa.

### Apical cell curvature

Apical cell contours were drawn manually from confocal images of meridional plans of apical cells immersed in NSW. Similar procedure was followed for tobacco pollen tubes from photos given by Gleb Grebnev (B. Kost’s lab, Erlangen Univ., Germany). We devised a Python 3 script to compute the average contour for a series of images and used it on *Ectocarpus* (*n* = 17; S2 Fig) and tobacco pollen tubes. The program starts with a hand-drawn contour for each cell, from which it computes a smoothed cubic spline curve. A set of equidistant points (we used a point-to-point distance of 50 nm) are extracted from the spline, and the meridional curvature κ_s_ is computed at each point (S2 Fig). To obtain average symmetrical curvatures, a pair of windows starting from the tip point and sliding in both directions was used (window width = 200 nm, sliding step = 50 nm). The discrete values of the κ_*s*_ = *f*(*s*) function were used to iteratively compute the position of cell wall point coordinates as values of *x* (the axial abscissa) and *r* (the distance to the axis), together with the meridional abscissa *s*, the curvatures κ_*s*_ and κ_θ_, and φ the angle between the axis and the normal to the cell wall. In particular, the circular symmetry of the dome imposes at the tip (where s = 0), that κ_θ_ = κ_s_ and thus σ_θ_ = σ_s_, whereas in the cylindrical part of the cell κ_s_ = 0 and thus σ_θ_ = 2σ_s_.

### Serial longitudinal sections of *Ectocarpus* apical cells

*Ectocarpus* filaments were prepared for TEM. Filaments grown on sterile glass slides were fixed with 4% glutaraldehyde and 0.25 M sucrose at room temperature and washed with 0.2 M sodium cacodylate buffer containing graded concentrations of sucrose. The samples were post-fixed in 1.5% osmium tetroxide, dehydrated with a gradient of ethanol concentrations, and embedded in Epon-filled BEEM capsules placed on the top of the algal culture. Polymerization was performed first overnight at 37 °C and then left for 2 d at 60 °C. Ultrathin serial sections were cut tangentially to the surface of the capsule with a diamond knife (ultramicrotome) and were mounted on copper grids or glass slides. Two types of sections were produced. Serial sections (300 nm thick) were stained with toluidine blue to show the main cellular structures, including the cell wall, and mounted on glass slides. Sections (70 nm thick) were stained with 2% uranyl acetate for 10 min and 2% lead citrate for 3 min, mounted on copper grids (Formvar 400 mesh; Electron Microscopy Science), and examined with a Jeol 1400 transmission electron microscope. A compilation of the sections for the 15 cells is shown in S3 Fig. Original photos are available at https://www.ebi.ac.uk/biostudies/studies/S-BSST215.

### Measurement of cell wall thickness and correction

From TEM pictures obtained on fixed *Ectocarpus* apical cells, only longitudinal sections with the thinnest walls were considered to avoid bias due to misaligned sections (all images are shown in S3 Fig). Measurements were carried out every 386 nm along 15 different cells, at the meridional abscissa from the tip (*s* = 0) up to *s* = ±70 μm using Fiji image analysis software. Altogether, 2,500 measured values of apparent thickness *w* were corrected, making the assumption that actual cell radius was *R* = 3.27 μm (but was seen as apparent radius *a*) and applying the following formula: $\delta =R-\sqrt{a^{2}+R^{2}-{\left(a+w\right)}^{2}}$ (S4 Data). As askew sectioning results in cell walls looking thicker, the only remaining bias is expected to cause overestimation of the thickness at the tip.

### Function for the meridional variation of the cell wall thickness

Corrected values δ for cell wall thickness were plotted as a function of the position *s* along the cell. As the relationship δ = *f*(*s*) displayed the aspect of an inverted bell, we designed 3 functions with this shape, derived from classical functions, to match them with the experimental values—(1) “Gauss”: $\delta =\delta_{max}-(\delta_{max}-\delta_{min})exp(-{\left(s/s_{1/2}\right)}^{2}log(2))$; (2) “Lorentz”: $\delta =\delta_{max}-(\delta_{max}-\delta_{min}){\left(1+{\left(s/s_{1/2}\right)}^{2}\right)}^{-1}$; and (3) “Pearson”: $\delta =\delta_{max}-(\delta_{max}-\delta_{min}){\left(1+3{\left(s/s_{1/2}\right)}^{2}\right)}^{-1/2}$. The values δ_min_, δ_max_, and *s*_1/2_ were adjusted for each of these functions, with a respective residual standard error of 0.08, 0.05, and 0.04. Therefore, we used the Pearson model with its optimized values δ_min_ = 36.2 nm, δ_max_ = 591 nm, and *s*_1/2_ = 16.81 μm for further modeling (Fig 5C).

### AFM

*Ectocarpus* cells were boiled twice in 1% SDS, 0.1 M EDTA and then treated with a solution of 0.5 M KOH at 100 °C. Pellet was rinsed extensively with MilliQ water and dried on a glass slide. Imaging was performed on dried samples. A Veeco Bioscope catalyst atomic force microscope coupled with a Zeiss inverted fluorescent microscope was used for imaging. RTESP probes (Bruker) were used in Scanasyst mode.

### Orthogonality of tip growth

The protocol was adapted from [30] and is described in detail in [31]. Young sporophyte filaments grown in glass-bottom petri dishes were covered with sonicated 0.1% (w:v NSW) of FluoSpheres amine, 0.2 μm, red (F8763, Molecular Probes), washed with NSW and mounted under a TCS SP5 AOBS inverted confocal microscope (Leica) controlled by the LASAF v2.2.1 software (Leica). The growth of 25 apical cells growing parallel to the glass surface was monitored, and bright-field and fluorescent pictures of median planes for each apical cell were acquired at several time points. Cell wall contours were hand-drawn on time-lapse images using GIMP, together with their respective indicator points. The position of the extreme tip (s = 0) was fixed for each meridional contour, and the drawing of cell contours and microsphere positions were aligned during the time course by using steady microspheres attached on fixed positions. A spline was adjusted on each contour and on each series of indicator points. The angle at each possible intersection between these trajectories and the cell contour splines were computed, making use of their first derivatives. Further analysis performed using R [78] consisted of (1) determining the distribution of angles, their mean, and standard deviation and (2) testing the hypothesis of dependence between the angle and the meridional abscissa. From the 156 measured angles between the tangent to cell wall and the trajectory, we computed the mean value *m* = 1.71 = π/1.83 radian (or π/2 − 9.16%) and the standard deviation *s* = 0.52 = π/6.09 radian. To test independence between the angle and the position in the dome, we computed the Pearson correlation coefficient between the angle and the absolute value of the meridional abscissa. It was *r* = −0.031.

### Calcofluor labeling

Staining of *Ectocarpus* filaments with calcofluor-white was carried out as described in [28].

### FM4-64 vesicle labeling and FRAP

FM4-64FX (F34653, Invitrogen) stock solution was diluted to 385 μM in DMSO and then diluted to 7.7 μM in NSW. Coverslips with *Ectocarpus* filaments were immersed in 50 μL of 7.7 μM cold FM4-64FX on ice and immediately mounted on a confocal microscope. Endocytosis and further trafficking of the fluorochrome was followed for 1 h at room temperature. The fluorochrome was excited with a 561 nm neon laser, and emission was observed with a 580–630 nm PMT.

For the FRAP assay, filaments were stained with 100 μM FM4-64FX for 10 min at 4 °C and rinsed 4 times with cold, fresh seawater. Photobleaching was performed on about 25 μm (*s*) along the cell from the tip, and recovery was monitored using an inverted Nikon Ti Eclipse Eclipse-E microscope coupled with a Spinning Disk (Yokogawa, CSU-X1-A1) and a FRAP module (Roper Scientifics, ILAS). Images were captured with a 100x APO TIRF objective (Nikon, NA 1.49) and an sCMOS camera (Photometrics, Prime 95B). For the detection of the FM4-64FX stained samples, we used a 488 nm laser (Vortran, 150 mW) for the excitation and the bleaching steps and collected the fluorescence through a 607/36 bandpass filter (Semrock). Image acquisition using the MetaMorph software 7.7 (Molecular Devices) was as follows: 1 image/s, displaying 6 images before bleaching, 1 image at the precise time of bleaching, 50 images during the recovery phase, for a total of 57 images by cell.

Images for one given cell were processed as a stack using Fiji [79] and R [78]. For each time point *t* (with bleaching occurring at *t* = 0), the background signal *Z*(*t*) was averaged from 4 separate square regions of approximately 1 μm^2^; the spontaneous fluorescence decrease was estimated by monitoring the signal *U*(*t*) in an unbleached region; the local signal was recorded in regions A–E as defined in Fig 9B. Note that all zones, including E, are sufficiently far from the edge of the photobleached zone to be unaffected by homogenization due to membrane lateral flux in the considered timescale. Following [80], the corrected signal for region A (and similarly for regions B–E) was computed as:

$$A_{c}(t)=(A(t)-Z(t)-(A(0)-Z(0)))\frac{U(0)-Z(0)}{U(t)-Z(t)}.$$

The recovery activity was estimated by matching the measured *A*_*c*_(*t*) values to the function *Y*(*t*) = *Y*(0) + *α*(1 − exp(−*t*/*τ*)), where *Y*(0) and *α* and *τ* are free parameters. We computed the normalized slope at *t* = 0 as (1/*α*)(d*A*_*c*_/d*t)*(0) = 1*/τ*, for 9 observations in each of the 5 (A–E) zones selected (see S7 Fig).

### Tobacco pollen tubes

The meridional contour of 6 tobacco pollen tube apices were traced from photos given by Gleb Grebnev (B. Kost’s group, Erlangen University, Germany), and the curvature was computed as described for *Ectocarpus* cells. Turgor and cell wall thickness were obtained from the literature [38]. In the absence of precise determination of their respective values, we derived a working hypothesis from previous literature reports showing that variations of Φ and σ_*y*_ occur simultaneously in opposite directions [49–51]. This intuitive relationship is consistent with molecular models of the cell wall [50]. Given that our model can derive the value of the expected strain rate ${\dot{\varepsilon }}^{*}$ from other values (S1 Text), we propose to partition this product equally between its two members. Thus, we computed $\Phi =\sqrt{{\dot{\varepsilon }}^{*}}$ and $(\sigma_{e}-\sigma_{y})=\sqrt{{\dot{\varepsilon }}^{*}}$, leading to $\sigma_{y}=\sigma_{e}-\sqrt{{\dot{\varepsilon }}^{*}}$. These arbitrary values were useful for giving an example of what could be a possible state (Fig 6F and 6G; Fig 7B right) and performing simulations.

### Code availability

Programs developed as part of this work were written in Python 3.6 [81], making use of NumPy [82] and Matplotlib [83] libraries, in a GNOME-Ubuntu environment (laptop and workstation). The source code is available at https://github.com/BernardBilloud/TipGrowth.

### Modeling and simulations

Modeling is described in S1 Text. The simulation program performed a simple simulation with graphic output or an array of simulations within a range of Φ and σ_y_ values. The input was a list of cell wall point coordinates and values from, for instance, computations of average contours (ad hoc generated data were also used for simulations starting with geometrically designed profiles). For each point, the stress was computed from turgor, curvature, and cell wall thickness values. Then, using Φ and σ_y_, the strain rate and the normal velocity were computed. The velocity and displacement direction (normal to the cell wall) gave the new position of the point, calibrated for a tip growth of 1 nm at each step. After computing new positions for all points, the program designed a cubic spline (without smoothing) from which a new sample of points was extracted, thus keeping a constant distance between points throughout the simulation. Accuracy of the simulation was evaluated by averaging point-to-point distances between the simulated profile and the initial profile translated at the expected speed. Values of Φ and σ_y_ were progressively optimized using a steepest-descent approach. As starting values, we used the coefficients of the linear model derived from the points (σ_e_,Φ(σ_e_ − σ_y_)) for which Φ(σ_e_ − σ_y_)) > 1: Φ = 2.5 × 10^−3^ min^−1^ MPa^−1^ and σ_y_ = 11 MPa. These values were used to simulate growth up to 25 μm, and divergence with the expected behavior was evaluated by comparing them to the initial points translated by 25 μm in the axial direction. As a numerical value, we took the logarithm of *rD* (residual distance), which was the weighted average point-to-point distance, where the weight was exp(*s*^2^log(2)), to maintain the dome shape. Optimized values Φ = 2.51 × 10^−3^ min^−1^ MPa^−1^ and σ_y_ = 11.18 MPa gave a simulation with a log(*rD*) of −3.0. As a comparison, the mean log(*rD*) between the initial average contour and the 17 experimental contours used to build it was −4.41, with a standard deviation of 0.35.

### Robustness

To assess the robustness of the results, we performed a bootstrap analysis. Three thousand samples were constructed by drawing with replacement 17 cell contours and 15 cell wall TEM images out of their respective datasets. For each sample, the average contour and the cell wall gradient were computed as explained above. The stress σ_e_ and expected strain rate ${\dot{\varepsilon }}^{*}$ were computed as functions of the meridional abscissa *s*. To test consistency with the model, the (σ_e_;${\dot{\varepsilon }}^{*}$) points were fitted a Lockhart equation by adjusting values Φ and σ_y_ and computing the Pearson correlation coefficient (*r*^2^) for the increasing part of the function, i.e., σ_e_ > σ_y_;${\dot{\varepsilon }}^{*}>0$.

## Supporting information

S1 Fig Cell surface deformation of *Ectocarpus* growing apical cells monitored by time-lapse fluorescent microscopy. The first 6 cells (first column, scale bar = 5 μm) were observed through an epifluorescence microscope; the other cells (second and third columns, scale bar = 5 μm) were observed through a confocal microscope. In each section, the first column represents bf pictures of apical cells at the beginning of the experiment (t0). The second column shows the corresponding fluorescent pictures (fluorescent microspheres attached to the cell surface), and the third column shows the cell meridional contour (blue) and positions of microspheres (red dots). Further time points (not similar for all cells) are shown in the next columns (t2 and t3). bf, bright-field.
(TIF)

S2 Fig Workflow for average contour computation. For each cell (grayscale image), a contour was manually drawn (superimposed pink line). This contour was smoothed (pink x-y plot), and local values of meridional curvature were computed (blue κ = f(s) plot). The 17 sets of values were averaged by a sliding window method, producing the average meridional curvature (blue plot in bottom right, standard deviation represented as light blue lines), which is eventually used to produce the average symmetric contour (pink plot). Data are available as S9 Data.
(TIF)

S3 Fig Longitudinal sections of apical cells observed by TEM. Sample of the 15 apical cells cut longitudinally and observed with several enlargements when necessary. Scale bars are indicated for each cell. Original photos are available at https://www.ebi.ac.uk/biostudies/studies/S-BSST215. TEM, transmission electron microscopy.
(TIF)

S4 Fig Robustness. Bootstrap analysis was used to assess the robustness of the major result of this paper. Three thousand replicates were generated by resampling over (1) the 17 cell contours and (2) the 15 series of cell wall thickness values. For each replicate, an average contour and cell wall gradient were computed. (A) Distribution for (left) minimum (at tip) and (center) maximum (asymptote) of the cell wall thickness gradient and (right) the correlation between these two values. There is a positive correlation because all samples exhibit a gradient (where, on the average, Δδ = 540 nm). (B) (Left) For each replicate, the expected strain rate was plotted against the stress. The grouping of curves displays a bundle aspect, showing that sampling preserves similarity to a Lockhart curve. (Center) This feature was confirmed by evaluating the linear adjustment of the increasing part of the curve (all points where σ_e_ > σ_y_) for each plot. The distribution of r^2^ is shown together with the curves displaying the lowest (0.682) and highest (0.999) r^2^. (Right) Plotting r^2^ against δ_min_ (and because of correlation between them, similarly for δ_max_) shows that, except for extreme values, r^2^ is not sensitive to δ_min_. (C) (Left and center) Distribution of plasticity values σ_y_ and Ф deduced from the previous curves and (right) correlation between them (note that scales for Ф are logarithmic). The positive correlation is coherent with the fact that curves in the panel B (left) tend to align or diverge rather than cross each other. In conclusion, throughout samples, the expected strain rate versus stress steadily exhibits a profile similar to a Lockhart curve, supporting the fact that σ_y_ and Ф are constant along the apical cell. These values vary among samples, and further studies would be necessary to determine them accurately. Data are available as S4 Data.
(TIF)

S5 Fig Cell wall isotropy in the apical cell. AFM pictures of cell wall ghosts extracted from the dome of an apical cell. (Left) View of the dome fully treated. (Middle) Close-up views. (Right) View of a dome not fully treated, showing naked cellulose microfibrils (and bundles) only in the bottom part and cellulose microfibrils embedded in the polysaccharide matrix in the top part. (Top) Relief of cellulose microfibrils/bundles. (Bottom) Peak-force energy. Note the random orientation of cellulose microfibrils (12.6 nm) and cellulose bundles (44 nm) arranged in several layers (the ghost cell comprises two cell wall layers). AFM, atomic force microscopy.
(TIF)

S6 Fig Simulation of tip growth with varying initial cell shapes (columns) and cell wall thickness gradients (rows). The impact of variations in initial cell shapes (“flat,” “*Ectocarpus*,” or “sharp”) was tested together with different cell wall thickness (δ) gradients (“steep,” “normal,” or “gentle”). (Left) Red curve is for normal gradient; gray curve is for modified gradient. (Right) Purple contour is for initial cell shape; blue contour is for final cell shape. Final stage of simulation is shown focused on the dome. Respective running simulations are shown in S4, S5 and S6 Movies.
(TIF)

S7 Fig FRAP protocol design and analysis. (A) top: Cell stained with FM4-64FX, before and after photobleaching. The bleached and unbleached regions are shown. (Bottom) Time course of a FRAP experiment: 1 image per second was taken for 6 s before the bleaching pulse, at the time of bleaching (t = 0 s), and during recovery (50 s). Images at t = −6 s, 0 s, 10 s, and 30 s for one cell are shown. (B) Fluorescent values used for normalizing the signal: the background was averaged from 4 random positions (signal shown as Back01 to Back04); the unbleached signal was taken from the cell wall in a region where no pulse has been applied. Normalization was performed as explained in Materials and methods. (C) Raw and corrected signal intensities taken from one region defined in Fig 5 are shown across time. The corrected signal intensity was used to adjust a theoretical recovery function I = I_0_(1 − exp(−t/τ)), for which the t_1/2_ is shown. The slope of this adjusted function at t = 0 was used as a proxy for the cell wall–building activity. FRAP, fluorescence recovery after photobleaching; t_1/2_, time for half recovery.
(TIF)

S1 Movie Tip-growth simulation in *Ectocarpus*. The cell wall, discretized using one point every 50 nm, was subjected to strain computed according to the viscoplastic model. Model parameters were deduced from observations, except yield threshold σ_y_ and extensibility Φ, which were optimized. The movie shows that these conditions allow reconstructing the expected growth process, i.e., dome expansion globally equivalent to translation at 2.5 μm h^−1^. The growth is followed for a simulated duration of 10 h. Green: initial shape; blue: simulated grown cell shape.
(MP4)

S2 Movie Effect of variations in cell wall yield threshold in *Ectocarpus*. Tip-growth simulations performed using measurements and optimized value for extensibility, and three different values for threshold: 10.18, 11.18, and 12.18 MPa. The central simulation uses the optimized threshold value σ_y_ = 11.18 MPa (identical to S1 Movie), and left and right simulations display the effect of a lower and higher value, respectively. The simulations ran synchronously up to 25 μm and show that changing yield threshold affects both growth rate and shape. Green: initial shape; purple: initial shape undergoing self-similar growth at the same speed as the simulated one; blue: simulated grown cell shape.
(MP4)

S3 Movie Effect of variations in cell wall extensibility in *Ectocarpus*. Tip-growth simulations performed using measurements and optimized value for yield threshold and three different values for extensibility: 1.51, 2.51, and 3.51 × 10^−3^ min^−1^ MPa^−1^. The central simulation uses the optimized extensibility value Φ = 2.51 × 10^−3^ min^−1^ MPa^−1^ (identical to S1 Movie), whereas left and right simulations display the effect of a lower and higher value, respectively. The simulations ran synchronously up to 25 μm showing that changing extensibility affects growth rate but not shape. Green: initial shape; blue: simulated grown cell shape.
(MP4)

S4 Movie Effect of variations in cell wall thickness gradient in *Ectocarpus*. Tip-growth simulations performed using optimized value for extensibility and yield threshold and measurements except cell wall thickness gradient: gentle, normal (i.e., that observed in *Ectocarpus*), and steep. The central simulation uses the measured gradient (identical to S1 Movie), whereas left and right simulations display the effect of a gentle and steep gradient, respectively. These altered gradients are obtained by changing the midpoint (s_1/2_) by 20%. The simulations ran synchronously up to 25 μm and show that changing the cell wall thickness gradient affects both growth rate and shape. Green: initial shape; purple: initial shape undergoing self-similar growth at the same speed as the simulated one; blue: simulated grown cell shape.
(MP4)

S5 Movie Effect of variations in initial cell shape in *Ectocarpus*. Tip-growth simulations performed using optimized value for extensibility and yield threshold but changing the initial cell shape: flat, “*Ectocarpus*,” and sharp. The middle simulation uses the normal *Ectocarpus* profile (identical to S1 Movie), whereas left and right simulations display the effect of a flat and sharp dome, respectively. These altered cell shapes were obtained by arbitrary computation. The simulations ran synchronously up to 25 μm growth and show that the different initial shapes quickly converge toward that of *Ectocarpus*. Green: initial shape; purple: initial shape undergoing self-similar growth at the same speed as the simulated one; blue: simulated grown cell shape.
(MP4)

S6 Movie Effect of change in cell wall thickness gradient and initial cell shape in *Ectocarpus*. Tip-growth simulations performed using measured and optimized value for all values except cell wall thickness and initial cell shape. The central simulation uses the *Ectocarpus* data (identical to S1 Movie), whereas other simulations display the effect of a flat or a sharp dome together with changes in cell wall thickness gradient. Movies focus on cell shape by displaying a close-up following the dome. Comparison of simulations shows that different initial profiles quickly converge toward a final shape constrained by the cell wall thickness gradient. Green: initial shape; purple: initial shape undergoing self-similar growth with the simulated tip; blue: simulated grown cell shape.
(MP4)

S1 Text Modeling and viscoplastic model parameters. (PDF)

S1 Data Angle between growth direction and cell wall. (XLSX)

S2 Data Measurement of turgor in the Ectocarpus apical cell using the limit plasmolysis method, and calculation of the correction due to shrinking. x = coefficient of volume variation after plasmolysis (ce = 1100 → 2660 mOsm.L-1) = V(plasmo)/V(initial) ci = internal osmolarity (mOsm.L-1) after volume variation ce = external osmolarity (mOsm.L-1) cpl = ce for which 50% of the cells are plasmolysed. Considered to be = ci if the cell variation is not taken into account Δc = difference between internal and external osmolarities (mOsm.L-1) T = turgor (MPa) = Δc/410.
(XLSX)

S3 Data Limit plasmolysis experiment. (XLSX)

S4 Data Cell wall thickness measurement and correction. (XLSX)

S5 Data Results of bootstrap analysis. (XLSX)

S6 Data Relative growth rate in different concentrations of Auxin (IAA). (XLSX)

S7 Data Measurement of turgor in the Ectocarpus apical cell treated with auxin IAA (similarly as in S2 Data). x = coefficient of volume variation after plasmolysis (ce = 1100 → 2660 mOsm.L-1) ci = internal osmolarity (mOsm.L-1) after volume variation ce = external osmolarity (mOsm.L-1) cpl = ce for which 50% of the cells are plasmolysed. Considered to be = ci if the cell variation is not taken into account Δc = difference between internal and external osmolarities (mOsm.L-1) T = turgor (MPa).
(XLSX)

S8 Data FRAP recovery rate at t = 0. (XLSX)

S9 Data Cell wall curvature. (XLSX)

## Abbreviations

AFM: atomic force microscopy
By: billion years
CER: chloroplastic endoplasmic reticulum
FRAP: fluorescence recovery after photobleaching
IAA: indole-3-acetic acid
My: million years
SEM: scanning electron microscopy
TEM: transmission electron microscopy
UDP: uridine diphosphate
WSC: cell wall integrity and stress response component
