# Supplementary figures and images for: The brown algal mode of tip growth: Keeping stress under control
Source: PLoS Biol. 2019 Jan 14;17(1):e2005258. doi: 10.1371/journal.pbio.2005258 (PMC6347293; doi:10.1371/journal.pbio.2005258)

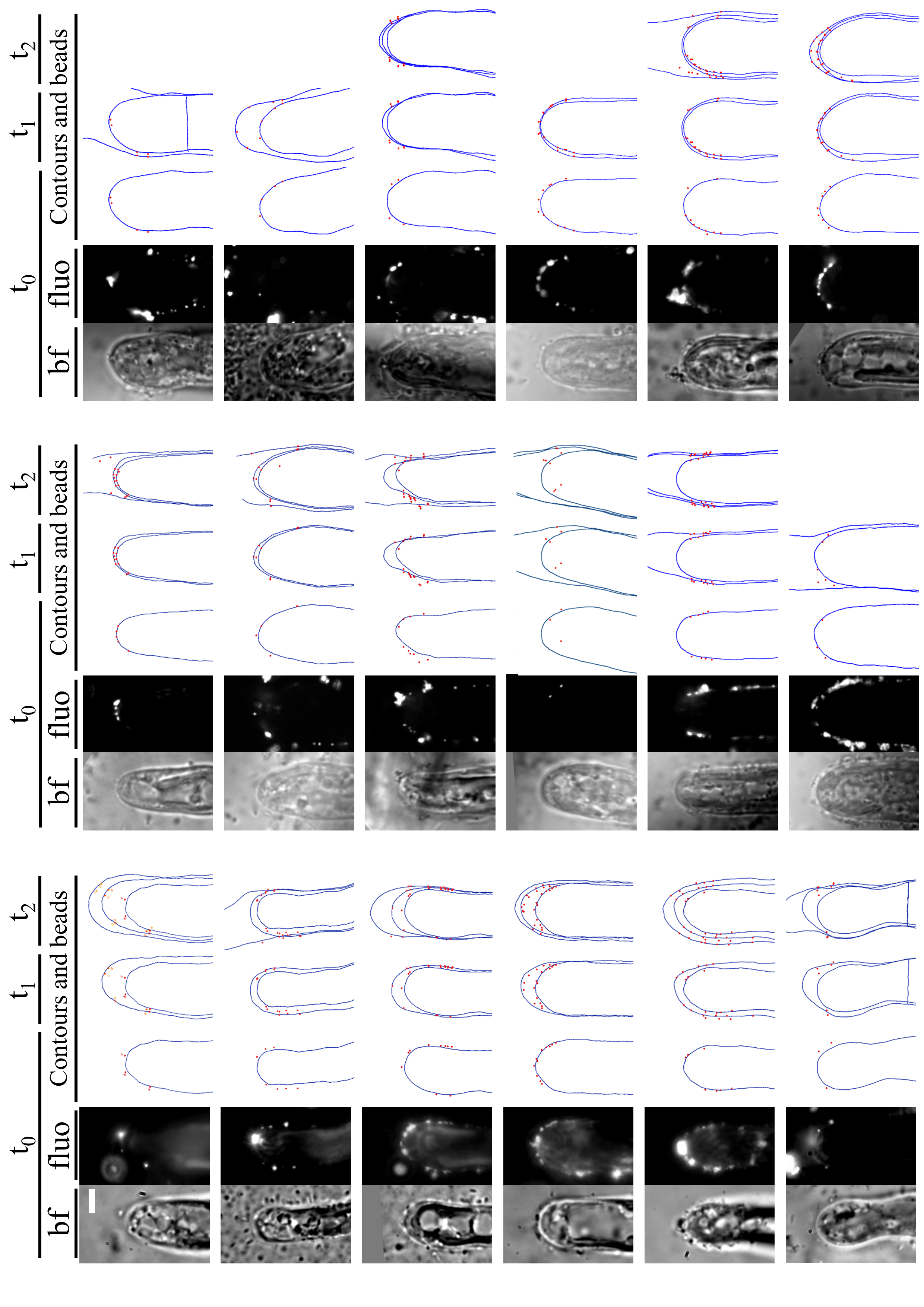

Supplement: S1 Fig — The first 6 cells (first column, scale bar = 5 μm) were observed through an epifluorescence microscope; the other cells (second and third columns, scale bar = 5 μm) were observed through a confocal microscope. In each section, the first column represents bf pictures of apical cells at the beginning of the experiment (t0). The second column shows the corresponding fluorescent pictures (fluorescent microspheres attached to the cell surface), and the third column shows the cell meridional contour (blue) and positions of microspheres (red dots). Further time points (not similar for all cells) are shown in the next columns (t2 and t3). bf, bright-field. (TIF) [file pbio.2005258.s001.tif]

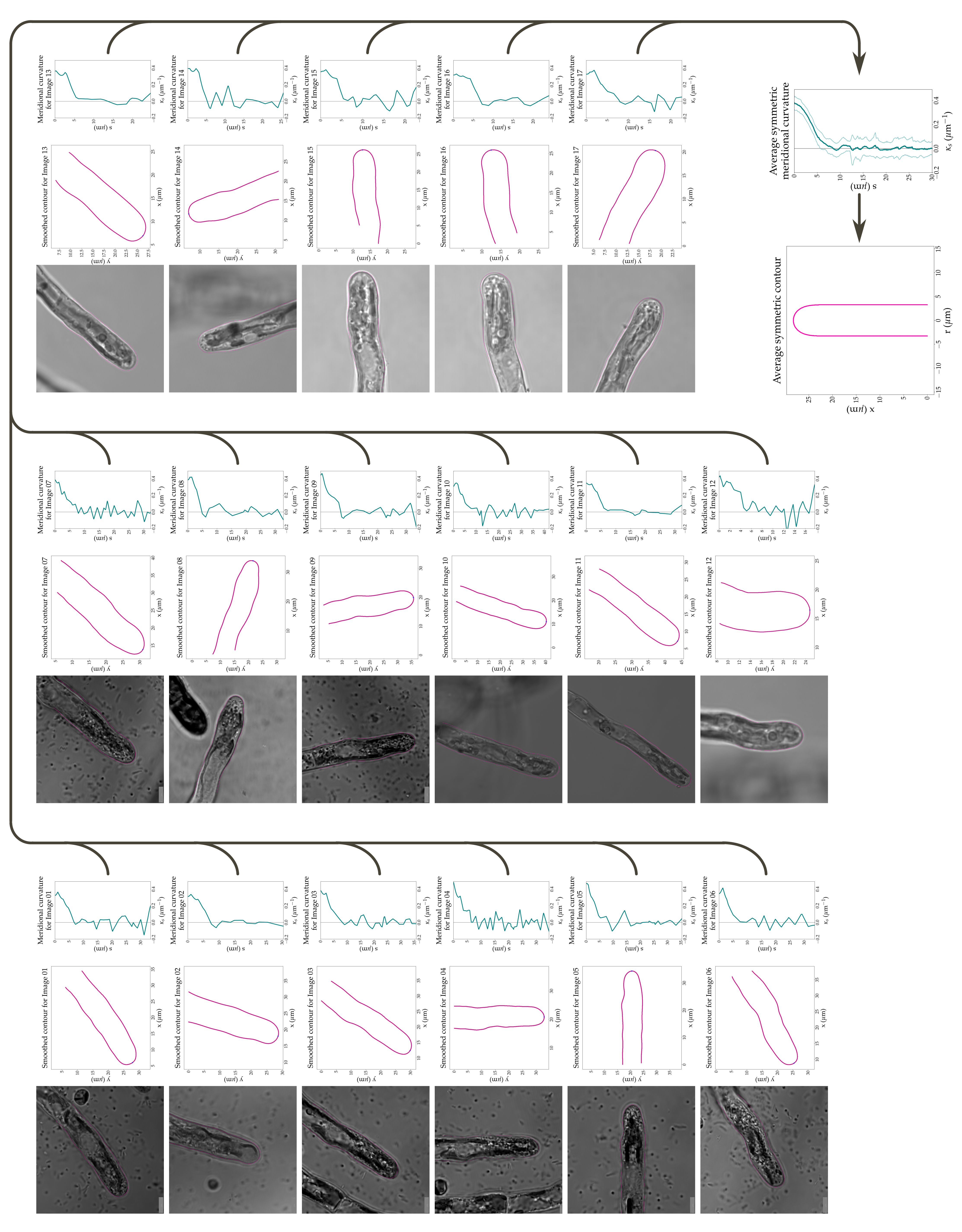

Supplement: S2 Fig — For each cell (grayscale image), a contour was manually drawn (superimposed pink line). This contour was smoothed (pink x-y plot), and local values of meridional curvature were computed (blue κ = f(s) plot). The 17 sets of values were averaged by a sliding window method, producing the average meridional curvature (blue plot in bottom right, standard deviation represented as light blue lines), which is eventually used to produce the average symmetric contour (pink plot). Data are available as S9 Data. (TIF) [file pbio.2005258.s002.tif]

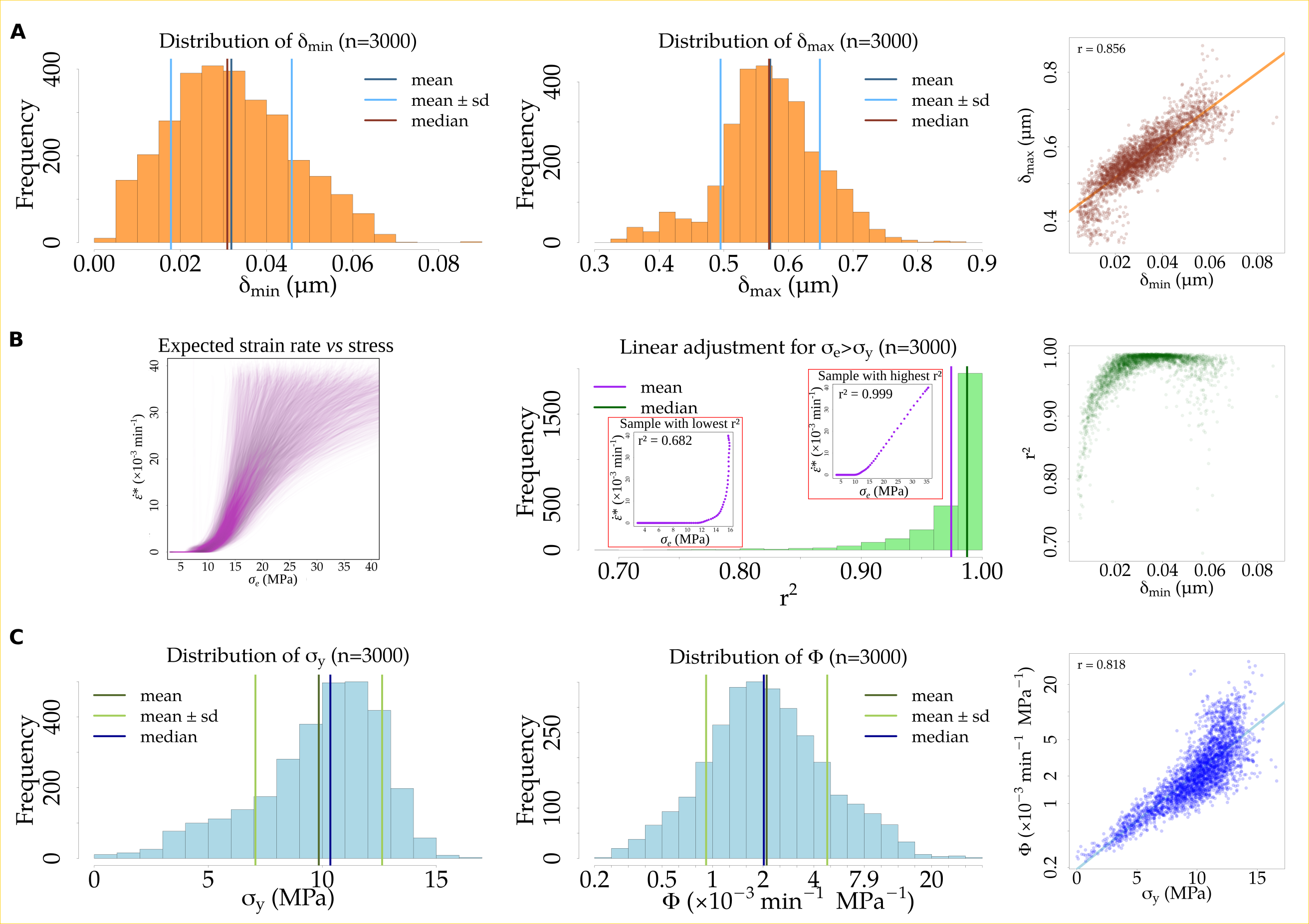

Supplement: S4 Fig — Bootstrap analysis was used to assess the robustness of the major result of this paper. Three thousand replicates were generated by resampling over (1) the 17 cell contours and (2) the 15 series of cell wall thickness values. For each replicate, an average contour and cell wall gradient were computed. (A) Distribution for (left) minimum (at tip) and (center) maximum (asymptote) of the cell wall thickness gradient and (right) the correlation between these two values. There is a positive correlation because all samples exhibit a gradient (where, on the average, Δδ = 540 nm). (B) (Left) For each replicate, the expected strain rate was plotted against the stress. The grouping of curves displays a bundle aspect, showing that sampling preserves similarity to a Lockhart curve. (Center) This feature was confirmed by evaluating the linear adjustment of the increasing part of the curve (all points where σe > σy) for each plot. The distribution of r2 is shown together with the curves displaying the lowest (0.682) and highest (0.999) r2. (Right) Plotting r2 against δmin (and because of correlation between them, similarly for δmax) shows that, except for extreme values, r2 is not sensitive to δmin. (C) (Left and center) Distribution of plasticity values σy and Ф deduced from the previous curves and (right) correlation between them (note that scales for Ф are logarithmic). The positive correlation is coherent with the fact that curves in the panel B (left) tend to align or diverge rather than cross each other. In conclusion, throughout samples, the expected strain rate versus stress steadily exhibits a profile similar to a Lockhart curve, supporting the fact that σy and Ф are constant along the apical cell. These values vary among samples, and further studies would be necessary to determine them accurately. Data are available as S4 Data. (TIF) [file pbio.2005258.s004.tif]

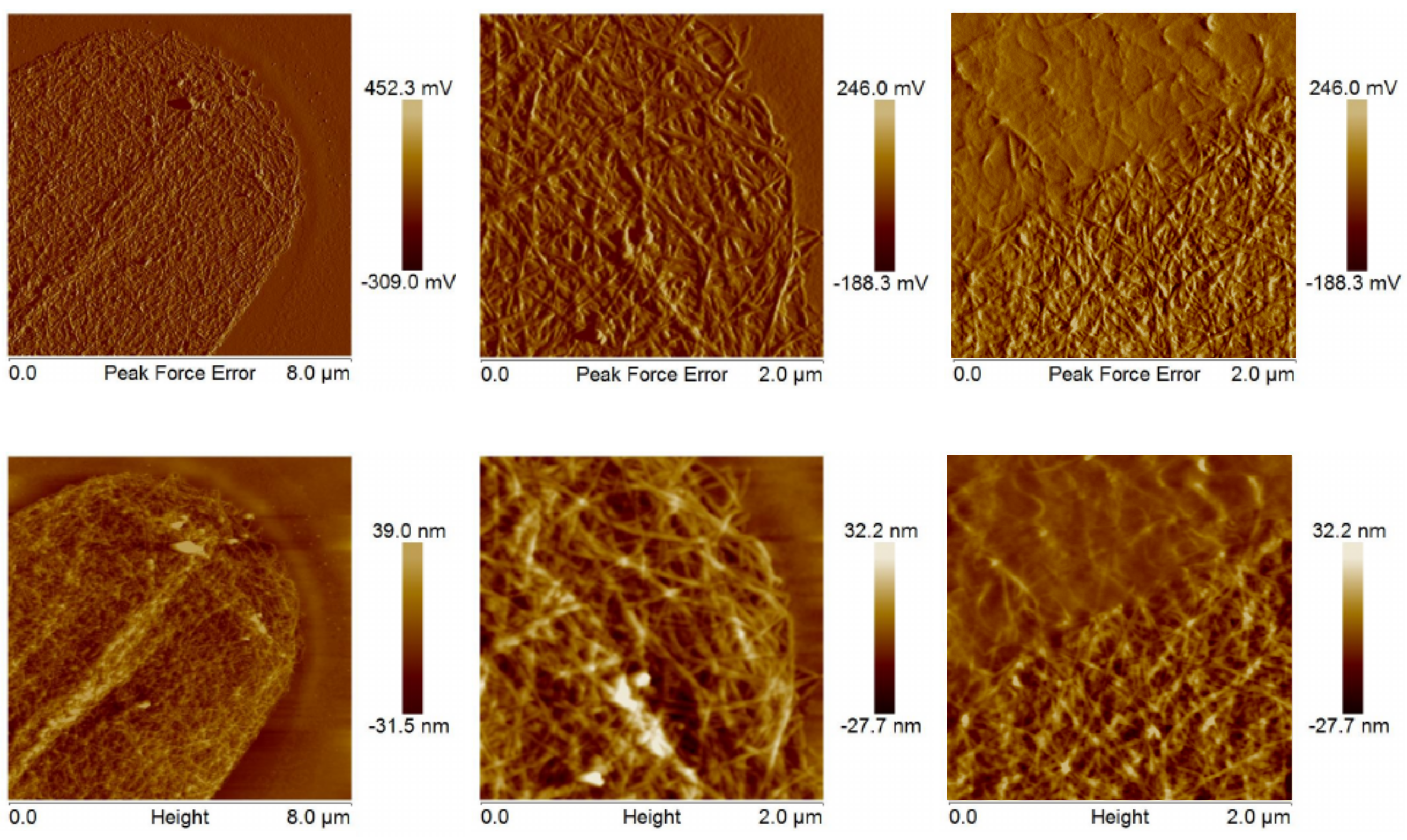

Supplement: S5 Fig — AFM pictures of cell wall ghosts extracted from the dome of an apical cell. (Left) View of the dome fully treated. (Middle) Close-up views. (Right) View of a dome not fully treated, showing naked cellulose microfibrils (and bundles) only in the bottom part and cellulose microfibrils embedded in the polysaccharide matrix in the top part. (Top) Relief of cellulose microfibrils/bundles. (Bottom) Peak-force energy. Note the random orientation of cellulose microfibrils (12.6 nm) and cellulose bundles (44 nm) arranged in several layers (the ghost cell comprises two cell wall layers). AFM, atomic force microscopy. (TIF) [file pbio.2005258.s005.tif]

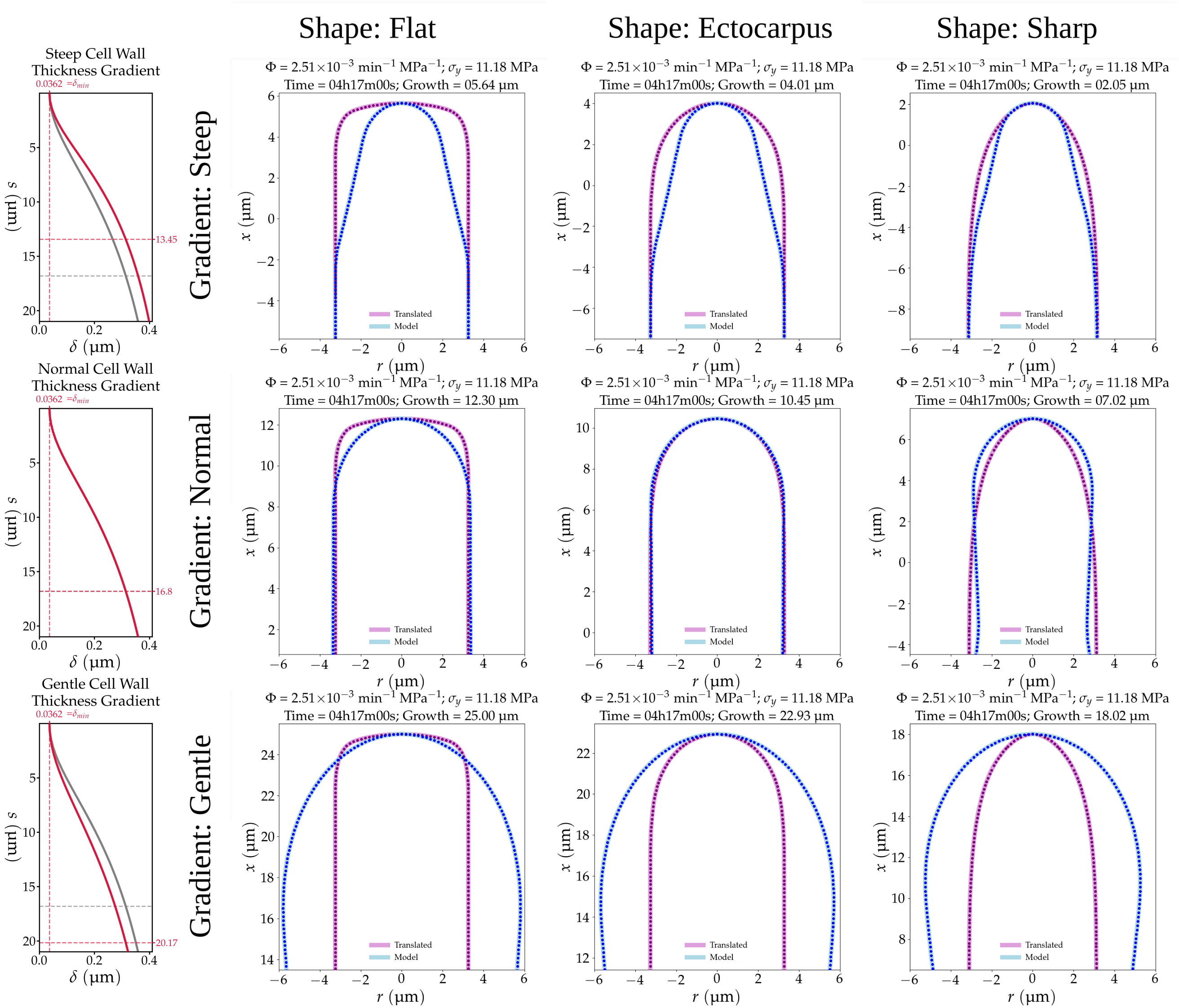

Supplement: S6 Fig — The impact of variations in initial cell shapes (“flat,” “Ectocarpus,” or “sharp”) was tested together with different cell wall thickness (δ) gradients (“steep,” “normal,” or “gentle”). (Left) Red curve is for normal gradient; gray curve is for modified gradient. (Right) Purple contour is for initial cell shape; blue contour is for final cell shape. Final stage of simulation is shown focused on the dome. Respective running simulations are shown in S4, S5 and S6 Movies. (TIF) [file pbio.2005258.s006.tif]

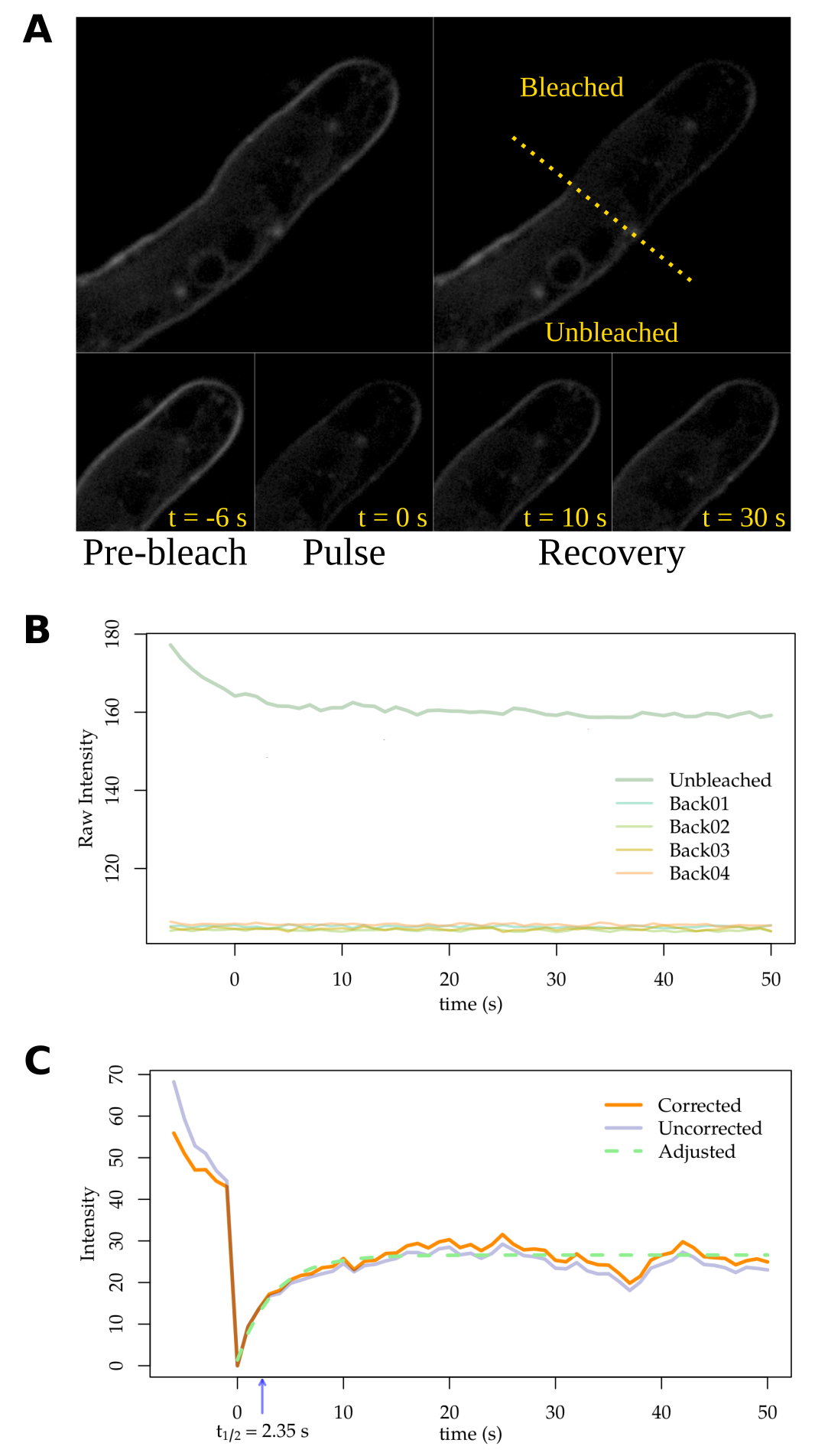

Supplement: S7 Fig — (A) top: Cell stained with FM4-64FX, before and after photobleaching. The bleached and unbleached regions are shown. (Bottom) Time course of a FRAP experiment: 1 image per second was taken for 6 s before the bleaching pulse, at the time of bleaching (t = 0 s), and during recovery (50 s). Images at t = −6 s, 0 s, 10 s, and 30 s for one cell are shown. (B) Fluorescent values used for normalizing the signal: the background was averaged from 4 random positions (signal shown as Back01 to Back04); the unbleached signal was taken from the cell wall in a region where no pulse has been applied. Normalization was performed as explained in Materials and methods. (C) Raw and corrected signal intensities taken from one region defined in Fig 5 are shown across time. The corrected signal intensity was used to adjust a theoretical recovery function I = I0(1 − exp(−t/τ)), for which the t1/2 is shown. The slope of this adjusted function at t = 0 was used as a proxy for the cell wall–building activity. FRAP, fluorescence recovery after photobleaching; t1/2, time for half recovery. (TIF) [file pbio.2005258.s007.tif]
