# Supplementary material for: The brown algal mode of tip growth: Keeping stress under control
Source: PLoS Biol. 2019 Jan 14;17(1):e2005258. doi: 10.1371/journal.pbio.2005258 (PMC6347293; doi:10.1371/journal.pbio.2005258)
Supplement: S1 Text — (PDF) [file pbio.2005258.s014.pdf]

Journal: PLoS Biology
Type: Research article, revision
Session: Biophysics

Title

The brown algal mode of tip growth: keeping stress under control.

Authors

Hervé Rabillé[1†], Bernard Billoud[1†], Benoit Tesson[2], Sophie Le Panse[3], Élodie Rolland[1], Bénédicte Charrier[1]*

[†]: these authors contributed equally to this work.

*: Benedicte.Charrier@sb-roscoff.fr

1: CNRS, Sorbonne Université, Morphogenesis of Macro Algae, UMR8227, Station Biologique, F-29680 Roscoff, France

2: SCRIPPS Institution of Oceanography, University of California, San Diego, CA 92093-0202, USA.

3: MerImage platform, FR2424, CNRS, Sorbonne Université, Station Biologique, F-29680 Roscoff, France

Supplementary information: modeling and viscoplastic model parameters

The law governing growth of a cylindrical cell subject to turgor pressure was established by Lockhart [1] using studies by Bingham [2]:

$$\begin{aligned} P \le Y &\Rightarrow \frac{1}{L}\dot{L} = 0 \\ P \ge Y &\Rightarrow \frac{1}{L}\dot{L} = R\,\Phi_L (P - Y) \end{aligned} \qquad \text{(S1)}$$

where L is the cell length (in µm), $\dot{L}$ the growth rate (in $\mu m \cdot h^{-1}$), R the radius (in µm), P the turgor pressure (in MPa), Y the yield threshold (in MPa), and Φ_L the wall extensibility (in $\mu m^{-1} \cdot h^{-1} \cdot MPa^{-1}$). The plasticity of the cell wall is driven by parameters Φ_L and Y, which, in equation (S1) behave as independent constant factors. Its graphic representation displays a characteristic appearance which is shown in Fig A.

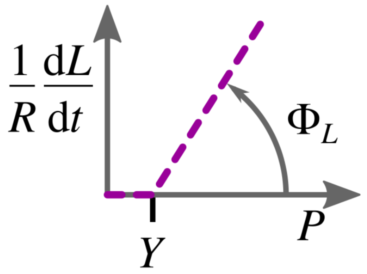

Fig A: Graphic representation of the Lockhart equation (S1).

Viscoplastic model of the cell wall

The viscoplastic model shows how the cell wall physical properties at microscopic level drive cell growth. This model focuses on the case of the apical cell in a filamentous organism. The cell is mostly cylindrical, with a dome-like tip, which is responsible for growth. Following Dumais et al. [3], this section considers an elementary portion of cell wall. Turgor pressure, acting on the whole cell wall, results in a local wall stress σ_e , which can vary from point to point. As the cell is supposed to admit a circular symmetry, σ_e only depends on the curvilinear abscissa s. This is the case for all variables, which should therefore be denoted as functions of s, but this will be usually omitted to keep notation simple. The small cell wall fragment expands if $\sigma_e > \sigma_y$, where σ_y is the yield threshold [4]. σ_y is a local cell wall property, which plays the same role as Y in the Lockhart equation (S1).

The stress can be partitioned into three directions: meridional (s), circumferential (θ) and normal (n). As the cell wall is thin compared to the cell dimensions, the normal component of the stress is considered negligible beside the two others [5]. Knowing the turgor pressure P, and having measured for each abscissa the cell wall thickness δ and the curvatures κ_s and κ_θ (Fig B), the local components of the stress are [6]:

$$\begin{aligned} \sigma_s &= \frac{P}{2\delta\kappa_\theta} \\ \sigma_\theta &= \frac{P}{2\delta\kappa_\theta}\left(2-\frac{\kappa_s}{\kappa_\theta}\right) \\ \sigma_n &= 0 \end{aligned} \qquad \text{(S2)}$$

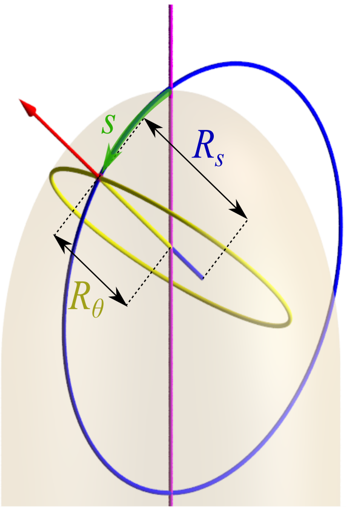

Fig B: Geometrical parameters of an apical cell: construction of the meridional (R_s, blue) and circumferential (R_θ, yellow) radii for a point (where the red arrow shows the normal to the surface) of curvilinear abscissa s: the circles are perpendicular to each other, and both are tangent to the cell wall.

Transverse isotropy

If the cell wall is transversely isotropic (*i.e.* the cell wall properties are identical in the meridional and circumferential directions), then the resulting value of global stress is:

$$\sigma_e = \sqrt{\nu\left(\sigma_\theta - \sigma_s\right)^2 + (1-\nu)\left(\sigma_\theta^2 + \sigma_s^2\right)} \tag{S3}$$

where ν is a dimensionless value denoting the flow coupling, equivalent to the Poisson ratio.

In these conditions, the cell wall part is subject to a strain whose rate is $\dot{\varepsilon}_e = \frac{1}{L}\frac{dL}{dt}$.

Plasticity is characterized by the relation between stress and strain rate:

$$\dot{\varepsilon}_e = \Phi\left(\sigma_e - \sigma_y\right) \tag{S4}$$

The global strain rate decomposes into its components according to:

$$\begin{cases} \dot{\varepsilon}_s = \Phi\left(\sigma_e - \sigma_y\right)\dfrac{\sigma_s - \nu\sigma_\theta}{K} \\ \dot{\varepsilon}_\theta = \Phi\left(\sigma_e - \sigma_y\right)\dfrac{\sigma_\theta - \nu\sigma_s}{K} \\ \dot{\varepsilon}_n = \Phi\left(\sigma_e - \sigma_y\right)\dfrac{(\nu - 1)\left(\sigma_s + \sigma_\theta\right)}{K} \end{cases} \tag{S5}$$

with:

$$K = \sqrt{2\left(\left(\nu^2 - \nu + 1\right)\left(\sigma_s^2 + \sigma_\theta^2\right) + \left(\nu^2 - 4\nu + 1\right)\sigma_s\sigma_\theta\right)} \tag{S6}$$

In these equations, Φ represents the local cell wall extensibility, which is the microscopic equivalent to the macroscopic extensibility Φ_L in Lockart's equation (S1).

The meridional and circumferential components of the strain rate induce an increase in cell wall surface, which is responsible for cell growth. The distribution of this increase between the two directions defines the local changes in cell shape which, taken together in the whole cell come out as global shape stability.

Orthogonal growth

If cell wall growth is orthogonal (*i.e.* each point of the wall moves perpendicular to the tangent to cell wall), then partitioning of growth between the meridional and circumferential axes becomes a direct consequence of cell shape:

$$\frac{\dot{\varepsilon}_\theta}{\dot{\varepsilon}_s}=\frac{\kappa_\theta}{\kappa_s} \tag{S7}$$

The flow coupling also illustrates this property:

$$\nu=\frac{1}{2}\left(1-\frac{\kappa_s}{\kappa_\theta}\right) \tag{S8}$$

At the tip of the dome, $\nu = 0$ while in the cylindrical part, $\nu = 1/2$.

In these conditions, the velocity of a point of the cell wall is:

$$V_n=\frac{\dot{\varepsilon}_\theta}{\kappa_\theta}=\frac{\dot{\varepsilon}_s}{\kappa_s} \tag{S9}$$

The viscoplastic model thus allows to understand the growth process at cell level as the integration of local strains resulting from geometrical and physical parameters, acting on infinitesimal parts of the cell wall.

Expected velocity and expected strain rate

Within the frame of the viscoplastic model, it is possible to set functions $\Phi(s)$ and $\sigma_y(s)$ which maintain insofar as possible the cell morphology during growth.

The target behavior of an apical cell development model is to grow without morphological change (Fig C). Locally, the dome is constantly reshaped, but at steady state the global result of the growth process is similar to a uniform translation along the longitudinal axis. Actually, under the hypothesis of orthogonal growth, each point of the dome moves in a direction that is normal to the cell wall. Over time, the local orientation of any part of the cell wall continuously shifts, so that the direction of point displacement changes. During an infinitesimal time step, however, the vector can be considered constant. By determining its direction and computing the intersection of this direction with the translated cell wall, it is possible to compute an expected normal velocity for each point, *i.e.* the function $V_n = f(s)$.

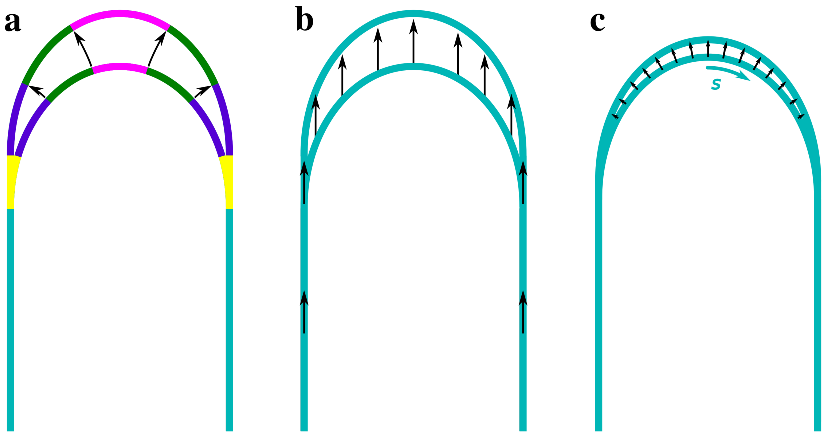

Fig C: **a,** A dome-like apex grows due to expansion of the cell wall in the dome region. **b,** Provided that this process maintains cell shape, it is globally equivalent to a uniform translation of the dome (self-similar tip growth, [7]), where the cylindrical part of the cell is considered of infinite length. **c,** For any point, the direction of the actual instantaneous velocity is normal to the cell wall, and its modulus is constrained by the necessity to maintain the global morphology of the cell.

As $\dot{\varepsilon}_\theta = \kappa_\theta V_n$ (eq. S9), rearranging (S5) and (S7) allows to express the local contribution to the cell deformation in the form of an expected strain rate:

$$\dot{\varepsilon}^* = \frac{K \kappa_\theta V_n}{\sigma_\theta - \nu \sigma_s} \qquad \text{(S10)}$$

At each point (that is: for each value of s), the values of κ_θ, V_n, σ_s, σ_θ, σ_e, ν and K are known: they can be deduced from the cell shape, cell wall width and turgor pressure (see above in the same paragraph, and equations S2, S3, S5, S6, S8). Therefore, $\dot{\varepsilon}^*(s)$ is computed without any knowledge about $\Phi(s)$ and $\sigma_y(s)$.

From expected strain rate to plasticity parameters

Under the assumptions of the viscoplastic model, the cell deformation results from local strain, *i.e.* the expected strain rate is nothing but the Lockhart strain rate, or:

$$\dot{\varepsilon}^*(s) = \dot{\varepsilon}_e(s) \qquad \text{(S11)}$$

Let us explicitly express the relation between stress and strain rate (equation S4) as functions of s:

$$\dot{\varepsilon}_e(s) = \Phi(s)\left(\sigma_e(s) - \sigma_y(s)\right) \qquad \text{(S12)}$$

This equation states that each value of the strain rate results from a local computation of the Lockhart function, where parameters may vary with s. Thus, the relation $\dot{\varepsilon}^* = f(\sigma_e)$ is expected to display a rather complex behavior reflecting changes in both stress and plasticity parameters, except in the very special situation when Φ and σ_y are

constant, in which case it reduces to the simple Lockhart function (graphic representation similar to Fig A).

Supplementary references

1. Lockhart JA. An analysis of irreversible plant cell elongation. J Theor Biol. 1965;8: 264–275. doi:10.1016/0022-5193(65)90077-9

2. Bingham EC. An investigation of the laws of plastic flow [Internet]. Washington, D.C.: U.S. Dept. of Commerce, Bureau of Standards : U.S. Govt. Print. Off.; 1917. Available: //catalog.hathitrust.org/Record/009487703

3. Dumais J, Shaw SL, Steele CR, Long SR, Ray PM. An anisotropic-viscoplastic model of plant cell morphogenesis by tip growth. Int J Dev Biol. 2006;50: 209–222. doi:10.1387/ijdb.052066jd

4. Hill R. The mathematical theory of plasticity. Clarendon Press; 1998.

5. Meyers MA, Chawla KK. Mechanical Behavior of Materials. 2nd ed. Cambridge ; New York: Cambridge University Press; 2008.

6. Hejnowicz Z, Heinemann B, Sievers A. Tip growth: Patterns of growth rate and stress in the Chara rhizoid. Z Für Pflanzenphysiol. 1977;81: 409–424. doi:10.1016/S0044-328X(77)80176-1

7. Goriely A, Tabor M. Mathematical modeling of hyphal tip growth. Fungal Biol Rev. 2008;22: 77–83. doi:10.1016/j.fbr.2008.05.001
